# Multiscale homogenization for dual porosity time-dependent Darcy–Brinkman/Darcy coupling and its application to the lymph node

**DOI:** 10.1098/rsos.231983

**Published:** 2024-07-17

**Authors:** A. Girelli, G. Giantesio, A. Musesti, R. Penta

**Affiliations:** ^1^ Dipartimento di Matematica e Fisica ‘N. Tartaglia’, Università Cattolica del Sacro Cuore, Brescia, Italy; ^2^ ‘Mathematics for Technology, Medicine and Biosciences’, Università degli Studi di Ferrara, Ferrara, Italy; ^3^ School of Mathematics and Statistics, University of Glasgow, Glasgow, UK

**Keywords:** mathematics, lymph node, multiscale, time-dependent, homogenization, Darcy–Brinkman

## Abstract

We study the coupling between time-dependent Darcy–Brinkman and the Darcy equations at the *microscale* subjected to inhomogeneous body forces and initial conditions to describe a double porosity problem. We derive the homogenized governing equations for this problem using the asymptotic homogenization technique, and as macroscopic results, we obtain a coupling between two Darcy equations, one of which with memory effects, with mass exchange between phases. The memory effects are a consequence of considering the time dependence in the Darcy–Brinkman equation, and they allow us to study in more detail the role of time in the problem under consideration. After the formulation of the model, we solve it in a simplified setting and we use it to describe the movement of fluid within a vascularized lymph node.

## 1. Introduction

The flow of fluids through porous media is a fundamental process with wide-ranging applications in various fields, such as hydrogeology and biology. In particular, porous media with dual porosity, where fluid flow occurs in different compartments with different pore structures, have garnered growing attention because of their widespread occurrence in both natural and engineered environments [1–7].

In this work, we present a multiscale model for dual-porosity porous media using the *asymptotic homogenization technique* [8,9] that couples a time-dependent Darcy–Brinkman equation [10] with a Darcy equation [8,9,11,12] which describes the fluid flow of the blood vessels inside the node, as our starting point. Here, we assume that the Darcy equation depends on time only parametrically. The theoretical justification of the Darcy–Brinkman equation is less warranted from a multiscale perspective compared to the Darcy equation [8,13–19]; however, since its structure is halfway between the Darcy and the Stokes equations, it can serve as a valid starting point for a multiscale formulation [1], and grants the ability to specify boundary conditions in more detail [20,21]. Moreover, the Darcy–Brinkman equation is also well defined in a time-dependent setting [14,22,23].

We consider a multiscale volume load that drives the coarse scale fluid flow acting on both the Darcy and Darcy–Brinkman problems [24]. These forces can occur from the use of electromagnetic fields, in magnetorheological fluids and in electrolytes that permeate non-uniform tissues [24]. Moreover, we consider a multiscale initial condition of the time-dependent problem that we take into account. Such initial conditions can arise for the flow of fluids in porous media with drug injection [25] or when as an initial condition we have another multiscale motion.

The derived model comprises porous media flow with memory effects (e.g. [8,26–29]), which are encoded in the effective hydraulic conductivity; thus, the nature of the obtained differential problem is intrinsically different with respect to [1]. The latter is represented by a convolution in time between the time-dependent hydraulic conductivity (obtained by solving the differential problem at the cell level) and the macroscopic pressure gradient. The macroscopic Darcy equation with memory effect that we obtain is new and differs from previous works on this subject such as [8,26–29] because we consider the effect of inhomogeneous body forces, a multiscale initial condition and the fluid exchange with another phase (described here by the Darcy equation). Using asymptotic homogenization, we pass from an equation at the microscale where *the present time is necessary for deducing the future* [30] to a macroscopic equation where *the present and the past are necessary to deduce the future* [31]. The memory effects appear to be a natural framework when dealing with the homogenization of this kind of problems [30,31].

Among the countless applications of dual-porosity porous media, the main motivation that guided us to propose and study this model is the application of the latter to the fluid flow of a lymph node, an essential part of the lymphatic system. The lymphatic system is composed of a network of vessels, capillaries and organs [32]. The interstitial fluid is drained by the *lymphatic capillaries* and it is called *lymph* once inside the lymphatic system. Inside the lymphatic vessels, there is a series of *one-way valves* which prevent retrograde flows. The part of the vessel between two valves is called *lymphangion*. Lymphangion walls are innervated with sympathetic and parasympathetic nerves [33–35] and can perform rhythmic contractions, making lymph transport a time-dependent flow [33,36–38]. The lymphatic system is an integral part of the immune system thanks to the *lymph nodes*. The lymph node is vital for immune defence, housing B and T cells that circulate in the body to protect against infection. B cells generate antibodies to fight antigens, while stimulated B cells transform into plasma or memory cells. Antigen-presenting cells capture antigens and present them to T cells in the lymph nodes, activating the adaptive immune response. Lymph transport has an important function in transporting immune cells, proteins, cancer metastasis, drugs and so on [39–41]. Furthermore, the movement of fluid through lymph nodes promotes the expression of chemokines, establishing a chemokine gradient that directs the movement of immune cells into the node. Changes in lymph transport play an important role in several pathologies. Elevated fluid flow augments the growth rate and sensitivity to drugs in specific forms of lymphomas [42]. Inadequate lymph transport can lead to a condition called lymphœdema, where excess interstitial fluid accumulates in the tissues, and it is often caused due to an impairment of lymph nodes [36,43]. From a mechanical perspective, the key characteristics of the lymph node include the lymphoid compartment (LC), which is a porous bulk region and forms the parenchyma of the lymph node, and the subcapsular sinus (SCS), a thin channel near the wall that surround the LC and that allows free-fluid flow [33]. The fluid can enter the LC from the SCS through a conduit system network created by fibroblastic reticular cells (FRCs) [44–46] which forms the main porous region of the lymph node. The lymph node is a highly vascularized organ, and inside the LC there are plenty of blood vessels that allow fluid and substance exchange [47–49], making the lymph node an important connection region between the lymphatic system and the blood system.

To the best of our knowledge, only a few models in the literature try to describe the fluid dynamical aspects of lymph node behaviour [50–52]. In [53,54], it is explored how the fluid flow in the lymph node is influenced by its internal structure, using an image-based model to establish a relationship between the greyscale values of the images and the permeability of the lymph node tissue, using this as the permeability for the steady Darcy equation. In [47], the authors conduct a parameter sensitivity analysis by using a computational flow model based on a mouse popliteal lymph node, coupling a steady Darcy–Brinkman equation in the LC with a steady Navier–Stokes equation in the SCS. Grebennikov and others [45,46] study the lymph flow through the conduit system network using an object-oriented computational algorithm to generate the three-dimensional geometry of the FRC graph network. In [55], the fluid flow within the lymph node is simulated by using a microfluidic device that mimics the microenvironment of a lymph node. Birmingham *et al*. [39] focused on the lymph node’s SCS fluid dynamics thanks to a microfluidic platform, evaluating how physiological flow patterns impact the adhesion of metastatic cancer cells (thanks to the wall shear stress values). In [56], a numerical method is developed to simulate the fluid flow in the lymph node using boundary integral equations, and then in [57] the authors provide an artificial neural network model to describe the lymph node drainage function. In [1,58,59], we have the first explicit models that describe the fluid flow in the lymph node in simplified geometries. In particular, in [58,59], they found a divergence-free explicit and numerical solution in a time-dependent setting, with a very idealized geometry for [58] and a spherical geometry in [59]. Girelli *et al*. [1] describe the blood vessel drainage function in the lymph node considering the multiscale nature of the latter in a steady setting, obtaining a rigorous mathematical model using the asymptotic homogenization technique; thanks to this approach, they were able to describe the fluid flow inside the conduit system network (formed by FRC) and inside the blood vessels networks.

This work addresses a crucial limitation characterizing the work in [1], as it takes into account the time-dependent character of the pulsatile flow which takes place in the lymph node due to the pulsation of the lymphangions [36,60,61] and its upscaling onto the macroscale. In fact, we present a multiscale model using the *asymptotic homogenization technique* [8,9] that couples a time-dependent Darcy–Brinkman equation [10], which describes the fluid flow inside the conduit system network formed by FRC, with a Darcy equation [8,9,11,12], which describes the fluid flow of the blood vessels inside the node, as our starting point. The Darcy equation depends on time only parametrically because the time-dependency we take into account is given by the lymphangion pulsation and affects mainly the lymph flow inside the FRC network. This model focuses on the porous region of the lymph node (LC) and the fluid exchange occurring solely between the node and the blood vessels within this specific area [47–49]. It is crucial to emphasize that, as highlighted in [1], our analysis begins with the Darcy–Brinkman and Darcy equations. This implies that variations in pore-scale geometry have been effectively averaged out, eliminating the necessity for precise details about the intricate and challenging-to-describe microstructure geometry of the lymph node.

In §2, we show the starting equations and boundary conditions of our problem, based on the balance equations of continuum mechanics. In §3, we employ the asymptotic homogenization technique and in §4, we find the macroscopic averaged equations. In §5, we find the explicit solution to our problem in spherical geometry under the hypothesis of axisymmetry with respect to the azimuthal angle and isotropy of the porous medium using the Fourier transform and using the explicit solution that we already found in [1]. In §6, we describe the numerical simulation that we use to solve the problem in the cell domain. Finally, in §7, we solve the explicit solution we found in §5 using lymph node physiological data obtained from the literature, allowing us to study in more detail the behaviour of the lymph inside the lymph node in a time-dependent setting.

## 2. Formulation of the starting problem

**Figure 1.**
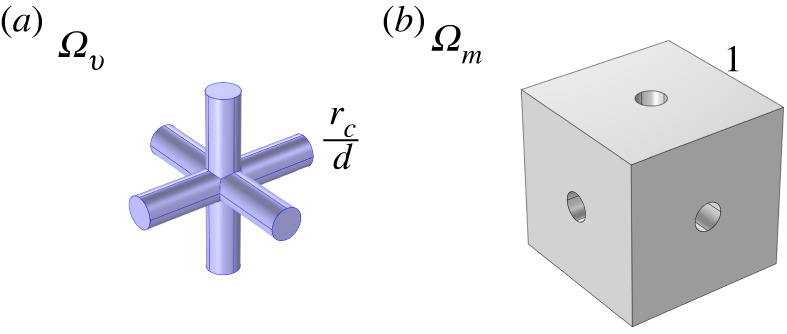
A sketch of a periodic portion of the domain comprising the vessels *Ω*_*v*_ (*a*) and matrix *Ω*_*m*_ (*b*).

We consider the domain $\Omega =\Omega_{v}\cup \Omega_{m}$, where *Ω*_*m*_ and *Ω*_*v*_ denote regions of the matrix and the vessels, respectively. A sketch of a portion of the three-dimensional domain at hand comprising the two phases is provided in figure 1. We describe the fluid flow in *Ω*_*v*_ via Darcy’s Law supplemented by a body force as in the work [24], namely

2.1 $$\left\{ \begin{array}{cc} v_{v}(x,t)=-{\tilde{K}}_{v}(x)(\nabla p_{v}(x,t)-b_{v}(x,t))\; & \text{in}\;\Omega_{v}\times [0,T]\;\\ \nabla \cdot v_{v}(x,t)=0\; & \text{in}\;\Omega_{v}\times [0,T].\; \end{array}\right.$$

In order to model the unsteady fluid flow within the matrix phase *Ω*_*m*_, we exploit the following *Darcy–Brinkman* equation [1,10,24]:

2.2 $$\left\{ \begin{array}{cc} \rho_{0}\frac{\partial v_{m}}{\partial t}(x,t)=-\nabla p_{m}(x,t)-{\tilde{K}}_{m}^{-1}(x)v_{m}(x,t)+\mu_{e}\Delta v_{m}(x,t)+b_{m}(x,t)\; & \text{in}\;\Omega_{m}\times [0,T]\;\\ \nabla \cdot v_{m}(x,t)=0\; & \text{in}\;\Omega_{m}\times [0,T].\;\; \end{array}\right.$$

For *γ* = *v*, *m*, $v_{\gamma }$ is the velocity of the fluid in the region $\Omega_{\gamma }$, $p_{\gamma }$ is the pressure in the region $\Omega_{\gamma }$, $b_{\gamma }$ is the inhomogeneous external force density in the region $\Omega_{\gamma }$, ${\tilde{K}}_{\gamma }(x)$ is the *hydraulic conductivity tensor* in the region $\Omega_{\gamma }$, *μ*_*e*_ is the *effective viscosity*, and *ρ*_0_ is the fluid *density*. For *γ* = *v*, *m*, we suppose that the hydraulic conductivity tensor is symmetric and positive definite:

2.3 $${\tilde{K}}_{\gamma }(x)={\tilde{K}}_{\gamma }^{T}(x),\;\forall \;a\neq 0\;:\;a\cdot {\tilde{K}}_{\gamma }(x)a>0.$$

Our starting point consists of both Darcy and Darcy–Brinkman equations, assuming that the pore structure is homogenized in both compartments. In this scenario, the hydraulic conductivity tensor ${\tilde{K}}_{\gamma }(x)$ functions as a means to represent the essential microscale geometric information.

The matrix and the vessels are coupled via the following interface conditions:

2.4 $$\left\{ \begin{array}{cc} v_{v}(x,t)\cdot n=v_{m}(x,t)\cdot n=L_{p}(p_{m}(x,t)-p_{v}(x,t)-\bar{\;p}(t))\; & \text{on}\;\Gamma \times [0,T]\;\\ v_{m}(x,t)\cdot \tau =-\frac{\sqrt{\mu {\tilde{K}}_{m}(x)}}{\alpha }[(n\cdot \nabla )v_{m}(x,t)]\cdot \tau \; & \text{on}\;\Gamma \times [0,T],\; \end{array}\right.$$

where $\Gamma =\partial \Omega_{m}\cap \partial \Omega_{v}$, ***n*** is the outer normal to *Ω*_*m*_, ***τ*** is any tangential vector to *Γ*, $\bar{\;p}(t)$ is a function that depends only on time and *α* is a constant that depends on the physico-chemical properties of the interface. The first interface condition of (2.4) is related to the normal component of the velocity; if we take $\bar{\;p}=\sigma (\pi_{m}-\pi_{v})$, we obtain the well-known *Starling equation* [62,63], used to describe the fluid exchange between two regions separated by a porous membrane, where *σ* is the *Staverman reflection coefficient*, *π*_*v*_ the *osmotic pressure of* *Ω*_*v*_ and *π*_*m*_ the *osmotic pressure of* *Ω*_*m*_. For simplicity, in this work, we assume that the osmotic pressures *π*_*v*_ and *π*_*m*_ depend only on time, but they can depend also on space [3]. The parameter *L*_*p*_ is typically experimentally measured and depends on the geometry and porosity/leakage of the vessels’ walls *Γ*. We consider this particular interface condition because we aim to apply this model to the fluid flow of a lymph node, but our formulation remains valid for other applications too. The second equation of (2.4) is the *Beavers–Joseph–Saffman boundary condition* [64], an interface condition on the tangent component of the velocity introduced in [65,66]. This interface condition introduces a slip velocity, which is proportional to the normal component of the velocity gradient of the fluid near the boundary. This interface condition is often used to describe the connection between free-fluid and porous regions, as well as an interface condition for dual-porosity media [2,67]. Moreover, linking the Darcy–Brinkman equation with the Darcy equation necessitates conditions reliant on the actual velocity. Therefore, the Beavers–Joseph conditions offer a more comprehensive grasp of the underlying physics compared to merely imposing a zero tangent velocity.

The initial condition is

2.5 $$v_{m}(x,0)=v_{m,0}(x)\;\text{in}\;\Omega_{m},$$

where ***v***_*m*,0_(***x***) need to satisfy $\nabla \cdot v_{m,0}(x)=0$ and must be compatible with the interface conditions (2.4).

The non-dimensional form of the Darcy equation, the Darcy–Brinkman equation, and the interface conditions for the domain $\Omega_{\gamma }$, with *γ* = *m*, *v*, are as follows. We denote with a prime symbol the following non-dimensional quantities:

2.6 $$t=\frac{L}{U}t^{\prime },\;v_{\gamma }=Uu_{\gamma }^{\prime },\;x=Lx^{\prime },\;{\tilde{K}}_{\gamma }=K_{\text{ref}}K_{\gamma }^{\prime },\;b_{\gamma }=\frac{U}{K_{\text{ref}}}b_{\gamma }^{\prime },\;p_{\gamma }=\frac{UL}{K_{\text{ref}}}p_{\gamma }^{\prime }$$

and

2.7 $$\epsilon =\frac{d}{L},$$

where *U* is the *characteristic velocity*, *K*_ref_ is the *representative (scalar) hydraulic conductivity*, *d* is the *fine-scale length* and *L* is the *coarse-scale length*. In the scope of this work, the parameter *d* takes on the role of denoting the separation between two vascularized regions. Instead of delving into the intricate particulars of individual vessels, the vascular network region is conceptualized as a geometric domain denoted as *Ω*_*v*_, consisting of interconnected cylinders with radius *r*_*c*_ (illustrated in figure 1), where the Darcy equation is applicable. Accordingly, *d* is precisely characterized as the distance between two neighbouring cylinders within this model representation.

Substituting (2.6) into (2.2), we obtain, by neglecting the primes

2.8 $$\left\{ \begin{array}{cc} \eta \frac{\partial v_{m}}{\partial t}(x,t)=-\nabla p_{m}(x,t)-K_{m}^{-1}(x)v_{m}(x,t)+\tilde{\mu }\Delta v_{m}(x,t)+b_{m}(x,t)\; & \text{in}\;\Omega_{m}\times [0,T],\;\\ \nabla \cdot v_{m}(x,t)=0\; & \text{in}\;\Omega_{m}\times [0,T],\; \end{array}\right.$$

where

$$\eta =\frac{\rho_{0}UK_{\text{ref}}}{L}\;\mathrm{and}\;\tilde{\mu }=\frac{K_{\text{ref}}\mu_{e}}{L^{2}}.$$

As *d* represents the fine-scale length in our problem, the most natural choice as a representative fine-scale conductivity, which we denote as *K*_ref_, resides, given its physical dimensions, in choosing $d^{2}/\mu$, i.e.

2.9 $$K_{\text{ref}}=\frac{d^{2}}{\mu },\;\eta =\frac{\rho_{0}Ud^{2}}{\mu L},\;\tilde{\mu }=\epsilon^{2}\mu^{*},\;\mu^{*}=\frac{\mu_{e}}{\mu }.$$

Substituting these relations into (2.1) and (2.8), we obtain the non-dimensional equations

2.10 $$\left\{ \begin{array}{cc} v_{v}(x,t)=-K_{v}(x)(\nabla p_{v}(x,t)-b_{v}(x,t))\; & \text{in}\;\Omega_{v}\times [0,T]\;\\ \nabla \cdot v_{v}(x,t)=0\; & \text{in}\;\Omega_{v}\times [0,T]\; \end{array}\right.$$

and

2.11 $$\left\{ \begin{array}{cc} \eta \frac{\partial v_{m}}{\partial t}(x,t)=-\nabla p_{m}(x,t)-K_{m}^{-1}(x)v_{m}(x,t)+\epsilon^{2}\mu^{*}\Delta v_{m}(x,t)+b_{m}(x,t)\; & \text{in}\;\Omega_{m}\times [0,T],\;\\ \nabla \cdot v_{m}(x,t)=0\; & \text{in}\;\Omega_{m}\times [0,T].\; \end{array}\right.$$

We also need to non-dimensionalize the interface conditions (2.4). In particular, we adopt the same distinguished limit embraced in [3, 68] which ensures that the blood flux stays finite when the length-scale separation that exists in the system becomes more and more pronounced. This is equivalent to assuming

2.12 $$\left\{ \begin{array}{cc} v_{v}(x,t)\cdot n=v_{m}(x,t)\cdot n=\epsilon {\bar{L}}_{p}(p_{m}(x,t)-p_{v}(x,t)-\bar{\;p}(t))\; & \text{on}\;\Gamma \times [0,T]\;\\ v_{m}(x,t)\cdot \tau =-\epsilon \frac{\sqrt{K_{m}(x)}}{\alpha }[(n\cdot \nabla )v_{m}(x,t)]\cdot \tau \; & \text{on}\;\Gamma \times [0,T],\; \end{array}\right.$$

where ${\bar{L}}_{p}=L_{p}\mu L^{2}/d^{3}$ [3]. Moreover, we close our problem using periodic boundary conditions at the boundary ∂Ω∖Γ.

## 3. The multiscale formulation

The goal of this section is to derive a macroscale model for the continuum system of equations (2.10), (2.5), (2.11) and (2.12), using the asymptotic homogenization technique [8,9,69]. We assume that there is a clear separation between the spatial fine scale *d* and the coarse scale *L*, which means that the quantity ϵ defined in (2.7) is small:

ϵ≪1.

To achieve spatial scale decoupling, we introduce a fresh local variable

3.1 $$y=\frac{x}{\epsilon },$$

where ***x*** represents the coarse-scale spatial coordinates and ***y*** represents the fine-scale spatial coordinates: they have to be considered independent in a formal way. We assume that $p_{\gamma }$, $v_{\gamma }$, $K_{\gamma }$ and $b_{\gamma }$ (where *γ* = *m*, *v*) depend on both ***x*** and ***y***.

We assume two main hypotheses concerning the geometry of the multiscale problem: *local periodicity* and *macroscopic uniformity*. Local periodicity means that $p_{\gamma }$, $v_{\gamma }$, $K_{\gamma }$ and $b_{\gamma }$ are ***y***-periodic. By making this assumption, we are able to focus our study on a restricted portion of the fine-scale domain. Instead, macroscopic uniformity means ignoring geometric variations within the cell structure and inclusions concerning the coarse-scale variable ***x***, *i.e.* the microstructure is unique. Then we may consider the utilization of a single periodic cell, denoted as *Ω*_*γ*_, for each macroscale point ***x***, and

3.2 $$\nabla_{x}\cdot \int_{\Omega_{\gamma }}(∙)dy=\int_{\Omega_{\gamma }}\nabla_{x}\cdot (∙)\;dy.$$

The differential operator becomes

3.3 $$\nabla \to \nabla_{x}+\frac{1}{\epsilon }\nabla_{y};$$

we now employ a power series expansion for ϵ for the variables of our problem as follows (where *γ* = *m*, *v*):

3.4 $$v_{\gamma }(x,y,t)\equiv v_{\gamma }^{\epsilon }(x,y,t)=\sum_{l=0}^{\infty }v_{\gamma }^{\left(l\right)}(x,y,t)\epsilon^{l},$$

3.5 $$p_{\gamma }(x,y,t)\equiv p_{\gamma }^{\epsilon }(x,y,t)=\sum_{l=0}^{\infty }p_{\gamma }^{\left(l\right)}(x,y,t)\epsilon^{l},$$

3.6 $$b_{\gamma }(x,y,t)\equiv b_{\gamma }^{\epsilon }(x,y,t)=\sum_{l=0}^{\infty }b_{\gamma }^{\left(l\right)}(x,y,t)\epsilon^{l}$$

3.7 $$\;\mathrm{and}\;v_{m,0}(x,y)\equiv v_{m,0}^{\epsilon }(x,y)=\sum_{l=0}^{\infty }v_{m,0}^{\left(l\right)}(x,y)\epsilon^{l}.$$

Substituting the asymptotic expansions (3.4)–(3.7) and the differential operator (3.3) into equations (2.10)–(2.11), we have

3.8 {ϵvvϵ(x,y,t)+ϵKv(x,y)∇xpvϵ(x,y,t)ϵvvϵ(x,y,t)+Kv(x,y)∇ypvϵ(x,y,t)−ϵKv(x,y)bvϵ(x,y,t)=0in Ωv×[0,T]ϵ∇x⋅vvϵ(x,y,t)+∇y⋅vvϵ(x,y,t)=0in Ωv×[0,T]

and

3.9 {ηϵ∂vmϵ∂t(x,y,t)=−ϵ∇xpmϵ(x,y,t)−∇ypmϵ(x,y,t)−ϵKm−1(x,y)vmϵ(x,y,t)ηϵ∂vmϵ∂t(x,y,t)=+μ∗ϵ3Δxvmϵ(x,y,t)+μ∗ϵΔyvmϵ(x,y,t)ηϵ∂vmϵ∂t(x,y,t)=+μ∗ϵ2∇x⋅(∇yvmϵ(x,y,t))+μ∗ϵ2∇y⋅(∇xvmϵ(x,y,t))ηϵ∂vmϵ∂t(x,y,t)=+ϵbmϵ(x,y,t)in Ωm×[0,T],ϵ∇x⋅vmϵ(x,y,t)+∇y⋅vmϵ(x,y,t)=0in Ωm×[0,T],

and if we substitute them into the interface conditions (2.12) and the initial condition (2.5), we obtain

3.10 $$\left\{ \begin{array}{cc} v_{v}^{\epsilon }(x,y,t)\cdot n=v_{m}^{\epsilon }(x,y,t)\cdot n=\epsilon {\bar{L}}_{p}(p_{m}^{\epsilon }(x,y,t)-p_{v}^{\epsilon }(x,y,t)-\bar{\;p}(t))\; & \text{on}\;\Gamma \times [0,T]\;\\ v_{m}^{\epsilon }(x,y,t)\cdot \tau =-\epsilon \frac{\sqrt{K_{m}(x,y)}}{\alpha }\left[\left(n\cdot \left(\nabla_{x}+\frac{1}{\epsilon }\nabla_{y}\right)\right)v_{m}^{\epsilon }(x,y,t)\right]\cdot \tau \; & \text{on}\;\Gamma \times [0,T]\; \end{array}\right.$$

and

3.11 $$v_{m}^{\epsilon }(x,y,0)=v_{m,0}^{\epsilon }(x,y)\;\text{in}\;\;\Omega_{m}.$$

### 3.1. Coefficients of order $\epsilon^{0}$

Collecting the terms of order $\epsilon^{0}$ from (3.8) to (3.11), we obtain

3.12 $$\nabla_{y}p_{v}^{\left(0\right)}(x,y,t)\Rightarrow p_{v}^{\left(0\right)}=p_{v}^{\left(0\right)}(x,t)\;\text{in}\;\Omega_{v}\times [0,T],$$

3.13 $$\nabla_{y}p_{m}^{\left(0\right)}(x,y,t)\Rightarrow p_{m}^{\left(0\right)}=p_{m}^{\left(0\right)}(x,t)\;\text{in}\;\Omega_{m}\times [0,T],$$

3.14 $$\nabla_{y}\cdot v_{v}^{\left(0\right)}(x,y,t)=0\;\text{in}\;\Omega_{v}\times [0,T],$$

3.15 $$\nabla_{y}\cdot v_{m}^{\left(0\right)}(x,y,t)=0\;\text{in}\;\Omega_{m}\times [0,T],$$

3.16 $$v_{m}^{\left(0\right)}(x,y,t)\cdot n=v_{v}^{\left(0\right)}(x,y,t)\cdot n=0\;\text{on}\;\Gamma \times [0,T],$$

3.17 $$v_{m}^{\left(0\right)}(x,y,t)\cdot \tau =-\frac{\sqrt{K_{m}(x,y)}}{\alpha }\left[(n\cdot \nabla_{y})v_{m}^{\left(0\right)}(x,y,t)\right]\cdot \tau \;\text{on}\;\Gamma \times [0,T]$$

3.18 $$\;\mathrm{and}\;v_{m}^{\left(0\right)}(x,y,0)=v_{m,0}^{\left(0\right)}(x,y)\;\text{in}\;\Omega_{m}.$$

### 3.2. Coefficients of order $\epsilon^{1}$

If we collect the terms of order $\epsilon^{1}$ from equations (3.8) to (3.11), we have

3.19 $$v_{v}^{\left(0\right)}(x,y,t)+K_{v}(x,y)\left(\nabla_{x}p_{v}^{\left(0\right)}(x,t)+\nabla_{y}p_{v}^{\left(1\right)}(x,y,t)-b_{v}^{\left(0\right)}(x,y,t)\right)=0\;\text{in}\;\Omega_{v}\times [0,T],$$

3.20 $$\begin{array}{rl} & \nabla_{x}\cdot v_{v}^{\left(0\right)}(x,y,t)+\nabla_{y}\cdot v_{v}^{\left(1\right)}(x,y,t)=0\;\text{in}\;\Omega_{v}\times [0,T], \end{array}$$

3.21 $$\begin{array}{rl} & \eta \frac{\partial v_{m}^{\left(0\right)}}{\partial t}(x,y,t)=-\nabla_{x}p_{m}^{\left(0\right)}(x,t)-\nabla_{y}p_{m}^{\left(1\right)}(x,y,t)-K_{m}^{-1}(x,y)v_{m}^{\left(0\right)}(x,y,t)\\ & \;+\mu^{*}\Delta_{y}v_{m}^{\left(0\right)}(x,y,t)+b_{m}^{\left(0\right)}(x,y,t)\;\text{on}\;\Omega_{m}\times [0,T], \end{array}$$

3.22 $$\nabla_{x}\cdot v_{m}^{\left(0\right)}(x,y,t)+\nabla_{y}\cdot v_{m}^{\left(1\right)}(x,y,t)=0\;\text{on}\;\Omega_{m}\times [0,T],$$

3.23 $$\begin{array}{rl} & v_{m}^{\left(1\right)}(x,y,t)\cdot n=v_{v}^{\left(1\right)}(x,y,t)\cdot n={\bar{L}}_{p}(p_{m}^{\left(0\right)}(x,t)-p_{v}^{\left(0\right)}(x,t)-\bar{\;p}(t))\;\text{on}\;\Gamma \times [0,T], \end{array}$$

3.24 $$v_{m}^{\left(1\right)}(x,\;y,\;t)\cdot \tau =-\frac{\sqrt{K_{m}(x,\;y)}}{\alpha }\left[(n\cdot \nabla_{x})v_{m}^{\left(0\right)}(x,\;y,\;t)+(n\cdot \nabla_{y})v_{m}^{\left(1\right)}(x,\;y,\;t)\right]\cdot \tau \;\text{on}\;\Gamma \times [0,\;T]$$

3.25 $$\mathrm{and}\;v_{m}^{\left(1\right)}(x,y,0)=v_{m,0}^{\left(1\right)}(x,y)\;\text{in}\;\Omega_{m}.$$

### 3.3. Derivation of Darcy’s equation

Applying the $\nabla_{y}\cdot$ operator to (3.19) and remembering equation (3.14), we obtain

3.26 $$\nabla_{y}\cdot [K_{v}(x,y)(\nabla_{x}p_{v}^{\left(0\right)}(x,t)+\nabla_{y}p_{v}^{\left(1\right)}(x,y,t)-b_{v}^{\left(0\right)}(x,y,t))]=0\;\text{in}\;\Omega_{v}\times [0,T];$$

it follows that the boundary condition (3.16) is

3.27 $$[K_{v}(x,y)(\nabla_{x}p_{v}^{\left(0\right)}(x,t)+\nabla_{y}p_{v}^{\left(1\right)}(x,y,t)-b_{v}^{\left(0\right)}(x,y,t))]\cdot n=0\;\text{on}\;\Gamma \times [0,T].$$

We note that the problem is linear and that $\nabla_{x}p_{v}^{\left(0\right)}$ is constant in ***y***; hence we formulate the following solution ansatz:

3.28 $$p_{v}^{\left(1\right)}(x,y,t)=h_{v}(x,y,t)\cdot \nabla_{x}p_{v}^{\left(0\right)}(x,t)+{\tilde{h}}_{v}(x,y,t).$$

The ansatz (3.28) solves the problem (3.26)–(3.27) (it is a solution up to a ***y***-constant function), provided that the auxiliary vector and scalar fields ***h***_*v*_ and ${\tilde{h}}_{v}$ solve the following cell problems:

3.29 $$\left\{ \begin{array}{cc} \nabla_{y}\cdot [\nabla_{y}h_{v}(x,y,t)K_{v}{\left(x,y\right)}^{T}]=-\nabla_{y}\cdot K_{v}{\left(x,y\right)}^{T}\;\; & \text{in}\;\Omega_{v}\times [0,T],\;\\ {}[\nabla_{y}h_{v}(x,y,t)K_{v}{\left(x,y\right)}^{T}]n=-K_{v}{\left(x,y\right)}^{T}n\; & \text{on}\;\Gamma \times [0,T]\; \end{array}\right.$$

and

3.30 $$\left\{ \begin{array}{cc} \nabla_{y}\cdot [K_{v}(x,y)\nabla_{y}{\tilde{h}}_{v}(x,y,t)]=\nabla_{y}\cdot [K_{v}(x,y)b_{v}^{\left(0\right)}(x,y,t)]\;\; & \text{in}\;\;\Omega_{v}\times [0,T],\;\\ {}[K_{v}(x,y)\nabla_{y}{\tilde{h}}_{v}(x,y,t)]\cdot n=[K_{v}(x,y)b_{v}^{\left(0\right)}(x,y,t)]\cdot n\; & \text{on}\;\Gamma \times [0,T].\; \end{array}\right.$$

To ensure the solution uniqueness, we need to impose that ${\left\langle h_{v}(x,y,t)\right\rangle }_{\Omega_{v}}=0$ and ${\left\langle {\tilde{h}}_{v}(x,y,t)\right\rangle }_{\Omega_{v}}=0$, where the average operator ${\left\langle (∙)\right\rangle }_{\Omega_{\gamma }}$ is defined as

3.31 $${\left\langle (∙)\right\rangle }_{\Omega_{\gamma }}=\frac{1}{\left|\Omega_{\gamma }\right|}\int_{\Omega_{\gamma }}(∙)\;dy.$$

Substituting the ansatz (3.28) into equation (3.19), we obtain the following Darcy’s equation:

3.32 $$\begin{array}{rl} v_{v}^{\left(0\right)}(x,y,t) & =-(K_{v}(x,y)+K_{v}(x,y){\left(\nabla_{y}h_{v}(x,y,t)\right)}^{T})\nabla_{x}p_{v}^{\left(0\right)}(x,t)\\ & \;-K_{v}(x,y)\nabla_{y}{\tilde{h}}_{v}(x,y,t)+K_{v}(x,y)b_{v}^{\left(0\right)}(x,y,t). \end{array}$$

### 3.4. Derivation of Darcy’s equation with memory

Putting together (3.21), (3.15), (3.16), (3.17) and (3.18) in *Ω*_*m*_, we obtain the following *auxiliary Darcy–Brinkman problem in* $\left(v_{m}^{\left(0\right)},p_{m}^{\left(1\right)}\right)$:

3.33 {η∂vm(0)∂t(x,y,t)=−∇xpm(0)(x,t)−∇ypm(1)(x,y,t)η∂vm(0)∂t(x,y,t)=−Km−1(x,y)vm(0)(x,y,t)+μ∗Δyvm(0)(x,y,t)η∂vm(0)∂t(x,y,t)=+bm(0)(x,y,t)in Ωm×[0,T],∇y⋅vm(0)(x,y,t)=0in Ωm×[0,T],vm(0)(x,y,t)⋅n=0on Γ×[0,T],vm(0)(x,y,t)⋅τ=−Km(x,y)α[(n⋅∇y)vm(0)(x,y,t)]⋅τon Γ×[0,T],vm(0)(x,y,0)=vm,0(0)(x,y)in Ωm.

We apply the Fourier transform defined by

3.34 $$F[\phi (t)]=\hat{\phi }(\omega )=\int_{-\infty }^{\infty }\phi (t)e^{-2\pi it\omega }\;dt\;$$

to system (3.33), and we obtain (note that the initial condition can be written as $\delta (t)v_{m,0}^{\left(0\right)}(x,y)$, where *δ*(*t*) is the *Dirac delta*)

3.35 $$\begin{array}{rl} & 2\pi i\omega \eta {\hat{v}}_{m}^{\left(0\right)}(x,y,\omega )=-\nabla_{x}{\hat{\;p}}_{m}^{\left(0\right)}(x,\omega )-\nabla_{y}{\hat{\;p}}_{m}^{\left(1\right)}(x,y,\omega )-K_{m}^{-1}(x,y){\hat{v}}_{m}^{\left(0\right)}(x,y,\omega )\\ & \;+\mu^{*}\Delta_{y}{\hat{v}}_{m}^{\left(0\right)}(x,y,\omega )+{\hat{b}}_{m}^{\left(0\right)}(x,y,\omega )+v_{m,0}^{\left(0\right)}(x,y)\;\text{in}\;\Omega_{m}\times (-\infty ,\infty ), \end{array}$$

3.36 $$\nabla_{y}\cdot {\hat{v}}_{m}^{\left(0\right)}(x,y,\omega )=0\;\text{in}\;\Omega_{m}\times (-\infty ,\infty ),$$

3.37 $${\hat{v}}_{m}^{\left(0\right)}(x,y,\omega )\cdot n=0\;\text{on}\;\Gamma \times (-\infty ,\infty )$$

3.38 $$\mathrm{and}\;\;\;{\hat{v}}_{m}^{\left(0\right)}(x,y,\omega )\cdot \tau =-\frac{\sqrt{K_{m}(x,y)}}{\alpha }[(n\cdot \nabla_{y}){\hat{v}}_{m}^{\left(0\right)}(x,y,\omega )]\cdot \tau \;\text{on}\;\Gamma \times (-\infty ,\infty ).$$

We have a linear problem and $\nabla_{x}{\hat{\;p}}_{m}^{\left(0\right)}$ depends on the macroscale only; hence we formulate an ansatz for the solution

3.39 $${\hat{\;p}}_{m}^{\left(1\right)}(x,y,\omega )=-{\hat{h}}_{m}(x,y,\omega )\cdot \nabla_{x}{\hat{\;p}}_{m}^{\left(0\right)}(x,\omega )+{\hat{\tilde{h}}}_{m}(x,y,\omega )$$

and

3.40 $${\hat{v}}_{m}^{\left(0\right)}(x,y,\omega )=-{\hat{Q}}_{m}(x,y,\omega )\nabla_{x}{\hat{\;p}}_{m}^{\left(0\right)}(x,\omega )+{\hat{\tilde{q}}}_{m}(x,y,\omega ).$$

We have that (3.39) and (3.40) are the unique solutions of the auxiliary Darcy–Brinkman problem (3.33) provided that the auxiliary second rank tensor ${\hat{Q}}_{\gamma }$, the auxiliary vectors ${\hat{h}}_{\gamma },{\hat{\tilde{q}}}_{\gamma }$ and the auxiliary scalar function ${\hat{\tilde{h}}}_{\gamma }$ solve the following cell problems:

3.41 {−2πiωηQ^m(x,y,ω)=−I+(∇yh^m(x,y,ω))T+Km−1(x,y)Q^m(x,y,ω)−2πiωηQ^m(x,y,ω)=−μ∗ΔyQ^m(x,y,ω)in Ωm×(−∞,∞),∇y⋅Q^m(x,y,ω)=0in Ωm×(−∞,∞),Q^mT(x,y,ω)n=0 on Γ×(−∞,∞),Q^mT(x,y,ω)τ=−Kmα[(∇yQ^mT(x,y,ω))n]τon Γ×(−∞,∞),

and

3.42 {2πiωηq~^m(x,y,ω)=−Km−1(x,y)q~^m(x,y,ω)+μ∗Δyq~^m(x,y)2πiωηq~^γ(x,y,ω)=−∇yh~^m(x,y,ω)+b^m(0)(x,y,ω)+vm,0(0)(x,y)in Ωm×(−∞,∞),∇y⋅q~^m(x,y,ω)=0in Ωm×(−∞,∞),q~^m(x,y,ω)⋅n=0on Γ×(−∞,∞),q~^m(x,y,ω)⋅τ=−Km(x,y)α[(n⋅∇y)q~^m(x,y,ω)]⋅τon Γ×(−∞,∞).

If we apply the inverse Fourier transform $F^{-1}$ to (3.41) and (3.42), we obtain

3.43 {η∂Qm∂t(x,y,t)=−Km−1(x,y)Qm(x,y,t)+μ∗ΔyQmT(x,y,t)η∂Qm∂t(x,y,t)=+δ(t)I−(∇yhm(x,y,t))Tin Ωm×[0,T],∇y⋅Qm(x,y,t)=0in Ωm×[0,T],QmT(x,y,t)n=0on Γ×[0,T],QmT(x,y,t)τ=−Kmα[∇yQmT(x,y,t)n]τon Γ×[0,T],Qm(x,y,0)=0in Ωm,

and

3.44 {η∂q~m∂t(x,y,t)=−Km−1(x,y)q~m(x,y,t)+μ∗Δyq~m(x,y,t)η∂q~m∂t(x,y,t)=−∇yh~m(x,y,t)+bm(0)(x,y,t)in Ωm×[0,T],∇y⋅q~m(x,y,t)=0in Ωm×[0,T],q~m(x,y,t)⋅n=0on Γ×[0,T],q~m(x,y,t)⋅τ=−Kmα[(n⋅∇y)q~m(x,y,t)]⋅τon Γ×[0,T],q~m(x,y,0)=vm,0(0)(x,y)in Ωm.

We note that system (3.44) gives a null result if the initial condition $v_{m,0}^{\left(0\right)}(x,y)$ and the multiscale force ***b***_*m*_ are both zero.

For system (3.43), we define the quantities

3.45 $${\bar{h}}_{m}(x,y,t)=\int_{-\epsilon_{2}}^{t}h_{m}(x,y,s)\;ds\;\mathrm{and}\;{\bar{Q}}_{m}(x,y,t)=\int_{-\epsilon_{2}}^{t}Q_{m}(x,y,s)\;ds,$$

where $\epsilon_{2}$ is a small number >0, so that

3.46 $$h_{m}(x,y,t)=\frac{\partial {\bar{h}}_{m}}{\partial t}(x,y,t),\;Q_{m}(x,y,t)=\frac{\partial {\bar{Q}}_{m}}{\partial t}(x,y,t);$$

substituting these equations into (3.43) and integrating over time, we have

3.47 {η∂Q¯m∂t(x,y,t)=−Km−1(x,y)Q¯m(x,y,t)η∂Q¯m∂t(x,y,t)=+μ∗ΔyQ¯m(x,y,t)+I−(∇yh¯m(x,y,t))Tin Ωm×[0,T],∇y⋅Q¯m(x,y,t)=0in  Ωm×[0,T],Q¯mT(x,y,t)n=0on Γ×[0,T],Q¯mT(x,y,t)τ=−Kmα[(∇yQ¯mT(x,y,t))n]τon Γ×[0,T],Q¯m(x,y,0)=0in Ωm.

Applying the inverse Fourier transform $F^{-1}$ to the ansatz (3.39)–(3.40), we obtain

3.48 $$\begin{array}{rl} v_{m}^{\left(0\right)}(x,y,t) & =-F^{-1}[{\hat{Q}}_{m}(x,y,\omega )\nabla_{x}{\hat{\;p}}_{m}^{\left(0\right)}(x,\omega )]+{\tilde{q}}_{m}(x,y,t)\\ & =-\int_{0}^{t}\frac{\partial {\bar{Q}}_{m}}{\partial t}(x,y,t-s)\nabla_{x}p_{m}^{\left(0\right)}(x,s)\;ds+{\tilde{q}}_{m}(x,y,t), \end{array}$$

which is in the form of a *Darcy equation with memory effects* [8,26,27].

By the same computations, we obtain

3.49 $$p_{m}^{\left(1\right)}(x,y,t)=-\int_{0}^{t}\frac{\partial {\bar{h}}_{m}^{T}}{\partial t}(x,y,t-s)\nabla_{x}p_{m}^{\left(0\right)}(x,s)\;ds+{\tilde{h}}_{m}(x,y,t).$$

Moreover, we need that ${\left\langle {\bar{h}}_{m}(x,y,t)\right\rangle }_{\Omega_{m}}=0$ and ${\left\langle {\tilde{h}}_{m}(x,y,t)\right\rangle }_{\Omega_{m}}=0$ to ensure the uniqueness of the solution, where ${\left\langle \cdot \right\rangle }_{\Omega_{m}}$ is the average operator defined in (3.31).

## 4. Derivation of the macroscopic model

We now apply the average operator (3.31)–(3.48)

$${\left\langle v_{m}^{\left(0\right)}(x,y,t)\right\rangle }_{\Omega_{m}}=-\int_{0}^{t}{\left\langle \frac{\partial {\bar{Q}}_{m}}{\partial t}(x,y,t-s)\right\rangle }_{\Omega_{m}}\nabla_{x}p_{m}^{\left(0\right)}(x,s)\;ds+{\left\langle {\tilde{q}}_{m}(x,y,t)\right\rangle }_{\Omega_{m}},$$

where ${\bar{Q}}_{m}$ and ${\tilde{q}}_{m}$ are computed by problems (3.47) and (3.44), respectively.

Applying the average operator (3.31) to equation (3.22), we obtain (using the macroscopic uniformity)

4.1 $$\nabla_{x}\cdot {\left\langle v_{m}^{\left(0\right)}(x,y,t)\right\rangle }_{\Omega_{m}}+{\left\langle \nabla_{y}\cdot v_{m}^{\left(1\right)}(x,y,t)\right\rangle }_{\Omega_{m}}=0,$$

where, by the divergence theorem and the interface conditions (3.23), we have, for the phase *Ω*_*m*_

4.2 $${\left\langle \nabla_{y}\cdot v_{m}^{\left(1\right)}(x,y,t)\right\rangle }_{\Omega_{m}}=\frac{1}{\left|\Omega_{m}\right|}\int_{\Omega_{m}}\nabla_{y}\cdot v_{m}^{\left(1\right)}(x,y,t)\;dy=\frac{{\bar{L}}_{p}S}{\left|\Omega_{m}\right|}[p_{m}^{\left(0\right)}(x,t)-p_{v}^{\left(0\right)}(x,t)-\bar{\;p}(t)],$$

and hence

4.3 $$\nabla_{x}\cdot {\left\langle v_{m}^{\left(0\right)}(x,y,t)\right\rangle }_{\Omega_{m}}=-\frac{{\bar{L}}_{p}S}{\left|\Omega_{m}\right|}[p_{m}^{\left(0\right)}(x,t)-p_{v}^{\left(0\right)}(x,t)-\bar{\;p}(t)].$$

For the Darcy problem on the phase *Ω*_*v*_, applying the average operator (3.31) to equation (3.32), we obtain

4.4 $$\begin{array}{rl} {\left\langle v_{v}^{\left(0\right)}(x,y,t)\right\rangle }_{\Omega_{v}} & =-{\left\langle K_{v}(x,y)+K_{v}(x,y){\left(\nabla_{y}h_{v}(x,y,t)\right)}^{T}\right\rangle }_{\Omega_{v}}\nabla_{x}p_{v}^{\left(0\right)}(x,t)\\ & \;-{\left\langle K_{v}(x,y)\nabla_{y}{\tilde{h}}_{v}(x,y,t)\right\rangle }_{\Omega_{v}}+{\left\langle K_{v}(x,y)b_{v}^{\left(0\right)}(x,y,t)\right\rangle }_{\Omega_{v}}. \end{array}$$

We apply the average operator and the divergence theorem to equation (3.20) and, following the same computations as before, we obtain

4.5 $$\nabla_{x}\cdot {\left\langle v_{v}^{\left(0\right)}(x,y,t)\right\rangle }_{\Omega_{v}}+{\left\langle \nabla_{y}\cdot v_{v}^{\left(1\right)}(x,y,t)\right\rangle }_{\Omega_{v}}=0,$$

and using the divergence theorem and the interface conditions (3.23)

4.6 $${\left\langle \nabla_{y}\cdot v_{v}^{\left(1\right)}(x,y,t)\right\rangle }_{\Omega_{v}}=\frac{1}{\left|\Omega_{v}\right|}\int_{\Omega_{v}}\nabla_{y}\cdot v_{v}^{\left(1\right)}(x,y,t)\;dy=-\frac{{\bar{L}}_{p}S}{\left|\Omega_{v}\right|}[p_{m}^{\left(0\right)}(x,t)-p_{v}^{\left(0\right)}(x,t)-\bar{\;p}(t)],$$

where we used the fact that ***n***_*v*_ = −***n***; hence

4.7 $$\nabla_{x}\cdot {\left\langle v_{v}^{\left(0\right)}(x,y,t)\right\rangle }_{\Omega_{v}}=\frac{{\bar{L}}_{p}S}{\left|\Omega_{v}\right|}[p_{m}^{\left(0\right)}(x,t)-p_{v}^{\left(0\right)}(x,t)-\bar{\;p}(t)].$$

The total coarse velocity ***u***_*C*_ is

4.8 $$\begin{array}{rl} u_{C}= & |\Omega_{m}|{\left\langle v_{m}^{\left(0\right)}(x,y,t)\right\rangle }_{\Omega_{m}}+|\Omega_{v}|{\left\langle v_{v}^{\left(0\right)}(x,y,t)\right\rangle }_{\Omega_{v}}\\ & =-|\Omega_{m}|\int_{0}^{t}{\left\langle \frac{\partial {\bar{Q}}_{m}}{\partial t}(x,y,t-s)\right\rangle }_{\Omega_{m}}\nabla_{x}p_{m}^{\left(0\right)}(x,s)\;ds+|\Omega_{m}|{\left\langle {\tilde{q}}_{m}(x,y,t)\right\rangle }_{\Omega_{m}}\\ & \;-|\Omega_{v}|{\left\langle K_{v}(x,y)+K_{v}(x,y){\left(\nabla_{y}h_{v}(x,y)\right)}^{T}\right\rangle }_{\Omega_{v}}\nabla_{x}p_{v}^{\left(0\right)}(x)\\ & \;-|\Omega_{v}|{\left\langle K_{v}(x,y)\nabla_{y}{\tilde{h}}_{v}(x,y)\right\rangle }_{\Omega_{v}}+|\Omega_{v}|{\left\langle K_{v}(x,y)b_{v}^{\left(0\right)}(x,y)\right\rangle }_{\Omega_{v}}. \end{array}$$

Substituting (4.1) into equation (4.3) and (4.4) into equation (4.7), we obtain

4.9 $$\begin{array}{rl} \nabla_{x}\cdot \left(\int_{0}^{t}{\left\langle \frac{\partial \bar{Q}}{\partial t}(x,y,t-s)\right\rangle }_{\Omega_{m}}\nabla_{x}p_{m}^{\left(0\right)}(x,s)\;ds\right) & =\nabla_{x}\cdot {\left\langle {\tilde{q}}_{m}(x,y,t)\right\rangle }_{\Omega_{m}}\\ & \;+\frac{{\bar{L}}_{p}S}{\left|\Omega_{m}\right|}[p_{m}^{\left(0\right)}(x,t)-p_{v}^{\left(0\right)}(x,t)-\bar{\;p}(t)] \end{array}$$

and

4.10 $$\begin{array}{rl} & \nabla_{x}\cdot \left({\left\langle K_{v}(x,y)+K_{v}(x,y){\left(\nabla_{y}h_{v}(x,y,t)\right)}^{T}\right\rangle }_{\Omega_{v}}\nabla_{x}p_{v}^{\left(0\right)}(x,t)\right)\\ & =\;-\nabla_{x}\cdot {\left\langle K_{v}(x,y)\nabla_{y}{\tilde{h}}_{v}(x,y,t)\right\rangle }_{\Omega_{v}}\\ & \;+\nabla_{x}\cdot {\left\langle K_{v}(x,y)b_{v}^{\left(0\right)}(x,y,t)\right\rangle }_{\Omega_{v}}-\frac{{\bar{L}}_{p}S}{\left|\Omega_{m}\right|}[p_{m}^{\left(0\right)}(x,t)-p_{v}^{\left(0\right)}(x,t)-\bar{\;p}(t)]. \end{array}$$

Equation (4.11) is the well-known diffusion problem related to the Darcy equation, where we can find additional terms that are related to the fluid exchange between phases and the multiscale forces [1,24]. When the multiscale force ***b***_*v*_ is zero, the unique solution ${\tilde{h}}_{v}(x,y,t)$ of the system (3.30) is zero, and in this case, equation (4.11) becomes the Darcy equation with fluid exchange between phases as derived and solved in [2,3,68]. On the other hand, equation (4.10) is the Darcy equation diffusion problem with memory effect, with additional terms related to the multiscale forces [24], the fluid exchange between phases and the multiscale initial condition. We note that if the multiscale force ***b***_*m*_ and the initial condition ***v***_*m*,0_ are both zeros, the unique solution ${\tilde{q}}_{m}$ of the system (3.44) is zero. Taking into account the time dependence of the problem, we obtain a very different model from the previous one [1–3,24,68]. However, even when ignoring the contributions related to the external volume loads and the initial condition, the final model that we have obtained differs from the one in [8,26] due to the coupling and the fluid exchange between the Darcy equation with memory effect and the classical Darcy equation and due to the Darcy–Brinkman type cell problem which needs to be solved to compute the hydraulic conductivity ${\left\langle \partial \bar{Q}/\partial t\right\rangle }_{\Omega_{m}}$ for the matrix compartment *Ω*_*m*_.

Now we dimensionalize equations (4.1), (4.3), (4.4) and (4.7). We write the equations in dimensional form because, with regard to their application to the lymph node, this makes it easier to interpret the results and compare them with other studies on the same topic. We have

4.11 $$|\Omega_{m}|=\frac{\left|\Omega_{m}^{\text{tot}}\right|}{\left|\Omega \right|},\;|\Omega_{v}|=\frac{\left|\Omega_{v}^{\text{tot}}\right|}{\left|\Omega \right|}\;\mathrm{and}\;\;S=\frac{S^{\text{tot}}d}{\left|\Omega \right|},$$

where |*Ω*| is the lymph node total volume, $\left|\Omega_{m}^{\text{tot}}\right|$ is the phase *m* total volume, $\left|\Omega_{v}^{\text{tot}}\right|$ is the phase *v* total volume and *S*^tot^ is the total vessel surface. Hence equations (4.1), (4.4), (4.5) and (4.8) are (in dimensional form)

4.12 $$\begin{array}{rl} & {\left\langle v_{m}^{\left(0\right)}(x,y,t)\right\rangle }_{\Omega_{m}}=-\frac{d^{2}}{\mu }\int_{0}^{t}{\left\langle \frac{\partial \bar{Q}}{\partial t}(x,y,t-s)\right\rangle }_{\Omega_{m}}\nabla_{x}p_{m}^{\left(0\right)}(x,s)\;ds\\ & \;+U{\left\langle {\tilde{q}}_{m,1}(x,y,t)\right\rangle }_{\Omega_{m}}+U{\left\langle {\tilde{q}}_{m,2}(x,y,t)\right\rangle }_{\Omega_{m}}, \end{array}$$

4.13 $$\begin{array}{rl} & \nabla_{x}\cdot {\left\langle v_{m}^{\left(0\right)}(x,y,t)\right\rangle }_{\Omega_{m}}=-\frac{L_{p}S^{\text{tot}}}{\left|\Omega_{m}^{\text{tot}}\right|}[p_{m}^{\left(0\right)}(x,t)-p_{v}^{\left(0\right)}(x,t)-\bar{\;p}(t)], \end{array}$$

4.14 $$\begin{array}{rl} & {\left\langle v_{v}^{\left(0\right)}(x,y,t)\right\rangle }_{\Omega_{v}}=-\frac{d^{2}}{\mu }{\left\langle K_{v}(x,y)+K_{v}(x,y){\left(\nabla_{y}h_{v}(x,y,t)\right)}^{T}\right\rangle }_{\Omega_{v}}\nabla_{x}p_{v}^{\left(0\right)}(x,t)\\ & \;-U{\left\langle K_{v}(x,y)\nabla_{y}{\tilde{h}}_{v}(x,y,t)\right\rangle }_{\Omega_{v}}+U{\left\langle K_{v}(x,y)b_{v}^{\left(0\right)}(x,y,t)\right\rangle }_{\Omega_{v}} \end{array}$$

4.15 $$\mathrm{and}\;\nabla_{x}\cdot {\left\langle v_{v}^{\left(0\right)}(x,y,t)\right\rangle }_{\Omega_{v}}=\frac{L_{p}S^{\text{tot}}}{\left|\Omega_{v}^{\text{tot}}\right|}[p_{m}^{\left(0\right)}(x,t)-p_{v}^{\left(0\right)}(x,t)-\bar{\;p}(t)].$$

## 5. Explicit solution

In this section, we explicitly solve the problems given in §4.

For simplicity, we suppose ***v***_*m*,0_ = **0**, ***b***_*m*_ = **0**, ***b***_*v*_ = **0**; hence the unique solution of problems (3.30) and (3.44) is zero. Moreover, we assume the isotropy of both the porous media, which means

5.1 $${\bar{K}}_{v}={\bar{K}}_{v}I,\;{\bar{K}}_{m}={\bar{K}}_{m}(t)I,$$

where ${\bar{K}}_{v}$ and ${\bar{K}}_{m}(t)$ correspond to $d^{2}/\mu {\left\langle K_{v}+K_{v}{\left(\nabla_{y}h_{v}\right)}^{T}\right\rangle }_{\Omega_{v}}$ and $d^{2}/\mu {\left\langle Q_{m}(y,t)\right\rangle }_{\Omega_{m}}$, respectively. We have that ${\bar{K}}_{v}$ is constant in space due to the geometry and the hypotheses used, and it is found solving the cell problem (3.29) using COMSOL Multiphysics (see §6 for more details about the numerical simulations of the cell problem). We have that the dimensional value for the vessel hydraulic conductivity *K*_*v*_*d*^2^/*μ* is computed using the Kozeny–Carman formula, i.e. $(1/c_{0}){\left(\left|\Omega_{v}^{\text{tot}}\right|/S^{\text{tot}}\right)}^{2}$; see table 1 and [1, appendix B] for more details. For phase *m*, due to the memory term that appears in equation (4.1), it is better to work in the frequency domain to find an explicit solution. Hence, applying the average operator (3.31) to the ansatz (3.40), we have in dimensional form

5.2 $${\left\langle {\hat{v}}_{m}^{\left(0\right)}(x,y,\omega )\right\rangle }_{\Omega_{m}}=-{\hat{\bar{K}}}_{m}(\omega )\nabla_{x}{\hat{\;p}}_{m}^{\left(0\right)}(x,\omega )=-\frac{d^{2}}{\mu }{\left\langle {\hat{Q}}_{m}(y,\omega )\right\rangle }_{\Omega_{m}}\nabla_{x}{\hat{\;p}}_{m}^{\left(0\right)}(x,\omega ).$$

**Figure 2.**
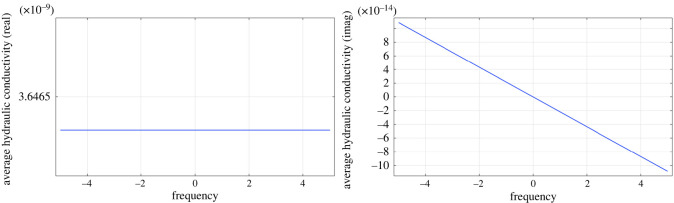
The Fourier transform of the dimensional average hydraulic conductivity in ${\text{mm}}^{3}\;\text{s}\;{\text{mg}}^{-1}$ calculated numerically with physiological values of the lymph node (table 1).

We find ${\hat{Q}}_{m}(y,\omega )$ by solving the cell problem (3.41) using COMSOL Multiphysics, with *α* = 1 (see §6 for more information about the numerical simulations of the cell problem and figure 2 for the results).

**Table 1.** Physiological and estimated parameters; see [1, appendix] for more details.

name	physiological range/value	description
*R*	0.49 mm	LC radius [39,59]
*μ*	$1\;\text{mg}\;{\mathrm{mm}}^{-1}{\text{ s}}^{-1}$	viscosity [36,37]
*ϕ*	0.75	porosity [55]
*μ*_*e*_	$\frac{\mu }{\phi }$	effective viscosity [10,70–72]
*ρ*_0_	$1\;\text{mg}\;{\text{mm}}^{-3}$	density [36,37]
\hat{\rm K}_m	3.84 × 10^−9^ mm^2^	interstitial permeability [46,55]
*σ*	0.88–0.9	Staverman’s coefficient [47,53,54,57]
*π*_*v*_ − *π*_*m*_	3.41 × 10^5^–2.08 × 10^6^ mPa	osmotic pressure difference [47,53,54,57,73–76]
*L*_*p*_	$5.475\times 10^{-12}\text{–}3.67\times 10^{-8}\;\text{mm}\;s^{-1}{\text{ mPa}}^{-1}$	hydraulic conductivity of the blood vessel walls [47,53,54,57]
${\bar{\;p}}_{v}$	6.67 × 10^5^–1.066 × 10^6^ mPa	mean blood vessel pressure [47,53,54,57]
*S*^tot^	13.4 mm^2^	surface of the blood vessels [48,49]
$\left|\Omega_{v}^{\text{tot}}\right|$	0.0322 mm^3^	volume of the blood vessel [48]
*N*	1310	cell number [1, appendix B]
*r*_*c*_	1.7 × 10^−3^ mm	cylinders radius (microscale) [1, appendix B]
*d*	2 × 10^−2^ mm	cylinder mean distance (microscale) [1, appendix B]
*L*	1 mm	characteristic length (macroscale)
*K*_*v*_ (*d*^2^/*μ*)	$1.1\times 10^{-6}\;{\text{mm}}^{3}\;\text{s}\;{\text{mg}}^{-1}$	blood vessels hydraulic conductivity, calculated using the Kozeny–Carman formula [1,77,78]
***b***_*m*_, ***b***_*v*_	**0**	body forces
${\hat{\bar{K}}}_{m}$	figure 2	macroscopic interstitial hydraulic conductivity (solving system (3.41))
${\bar{K}}_{v}$	$4.12\times 10^{-7}\;{\text{mm}}^{3}\;\text{s}\;{\text{mg}}^{-1}$	macroscopic blood hydraulic conductivity (solving system (3.29))

As we are working in the frequency domain, we apply the Fourier transform (3.34) to (4.9)–(4.9). We consider a spherical domain *Ω* with radius *R*, denoting by *r* the radial coordinate, *θ* the polar coordinate and *ϕ* the azimuthal angle. Moreover, we assume axisymmetry with respect to the azimuthal angle *ϕ*. Hence, we obtain the following problem:

5.3 $$\left\{ \begin{array}{cc} \Delta {\hat{\;p}}_{v}^{\left(0\right)}(r,\theta ,\omega )=-M_{v}[{\hat{\;p}}_{m}^{\left(0\right)}(r,\theta ,\omega )-{\hat{\;p}}_{v}^{\left(0\right)}(r,\theta ,\omega )-\bar{\;p}(\omega )]\; & r<R,\;\theta \in [0,2\pi [,\;\omega \in (-\infty ,\infty ),\;\\ \Delta {\hat{\;p}}_{m}^{\left(0\right)}(r,\theta ,\omega )=M_{m}(\omega )[{\hat{\;p}}_{m}^{\left(0\right)}(r,\theta ,\omega )-{\hat{\;p}}_{v}^{\left(0\right)}(r,\theta ,\omega )-\bar{\;p}(\omega )]\; & r<R,\;\theta \in [0,2\pi [,\;\omega \in (-\infty ,\infty ),\;\\ {\hat{\;p}}_{v}^{\left(0\right)}(R,\theta ,\omega )={\hat{\bar{\;p}}}_{v}(\theta ,\omega ),\;{\hat{\;p}}_{m}^{\left(0\right)}(R,\theta ,\omega )={\hat{\bar{\;p}}}_{m}(\theta ,\omega )\; & \theta \in [0,2\pi [,\;\omega \in (-\infty ,\infty ),\;\\ \text{non-degeneracy}\; & r=0,\;\theta \in [0,2\pi [,\;\omega \in (-\infty ,\infty ),\; \end{array}\right.$$

where *R* is the sphere radius, $M_{v}=L_{p}S^{\text{tot}}/|\Omega_{v}^{\text{tot}}|{\bar{K}}_{v}$, $M_{m}(\omega )=L_{p}S^{\text{tot}}/\left|\Omega_{m}^{\text{tot}}|{\hat{\bar{K}}}_{m}(\omega \right)$, and ${\hat{\bar{\;p}}}_{v}$ and ${\hat{\bar{\;p}}}_{m}$ are the Fourier transforms of the given boundary condition.

Following the computations presented in [1, appendix A], we obtain the following solutions (see [1, appendix A]):

5.4 $${\hat{\;p}}_{m}^{\left(0\right)}(r,\zeta ,\omega )=\sum_{n=0}^{\infty }\left[c_{1}^{\left(n\right)}(\omega )r^{n}+\frac{M_{m}(\omega ){\tilde{A}}_{n}(\omega )}{M(\omega )}\frac{1}{\sqrt{r}}I_{n+(1/2)}\left(\sqrt{M(\omega )}r\right)\right]P_{n}(\zeta )$$

and

5.5 $${\hat{\;p}}_{v}^{\left(0\right)}(r,\zeta ,\omega )=\sum_{n=0}^{\infty }\left[d_{1}^{\left(n\right)}(\omega )r^{n}-\frac{M_{v}{\tilde{A}}_{n}(\omega )}{M(\omega )}\frac{1}{\sqrt{r}}I_{n+(1/2)}\left(\sqrt{M(\omega )}r\right)\right]P_{n}(\zeta ),$$

where ζ=cos⁡θ, *M*(*ω*) = *M*_*v*_ + *M*_*m*_(*ω*), *I*_*n*_ is the modified Bessel function of the first kind of order *n*, *P*_*n*_ is the Legendre polynomial of the first kind of order *n*, and (the following relations are obtained from [1, appendix A])

5.6 $${\tilde{A}}_{0}(\omega )=\frac{[b^{\left(0\right)}(\omega )-\bar{\;p}(\omega )]\sqrt{R}}{I_{\left(1/2\right)}\left(\sqrt{M(\omega )}R\right)}\;\text{\textbackslash{},for}\;n=0,\;{\tilde{A}}_{n}(\omega )=\frac{b^{\left(n\right)}(\omega )\sqrt{R}}{I_{n+(1/2)}\left(\sqrt{M(\omega )}R\right)}\;\text{ for}\;n\geq 1,$$

5.7 $$c_{1}^{\left(n\right)}(\omega )=\frac{\left[b_{m}^{\left(n\right)}(\omega )-(M_{m}(\omega ){\tilde{A}}_{n}(\omega )/M(\omega )\sqrt{R})I_{n+(1/2)}\left(\sqrt{M(\omega )}R\right)\right]}{R^{n}},$$

5.8 $$d_{1}^{\left(n\right)}(\omega )=\frac{\left[b_{v}^{\left(n\right)}(\omega )+(M_{v}{\tilde{A}}_{n}(\omega )/M(\omega )\sqrt{R})I_{n+(1/2)}\left(\sqrt{M(\omega )}R\right)\right]}{R^{n}},$$

5.9 $$b^{\left(n\right)}(\omega )=\frac{1}{2}(2n+1)\int_{-1}^{1}[{\hat{\bar{\;p}}}_{m}(\zeta ,\omega )-{\hat{\bar{\;p}}}_{v}(\zeta ,\omega )]P_{n}(\zeta )d\zeta$$

5.10 $$\mathrm{and}\;b_{m}^{\left(n\right)}(\omega )=\frac{1}{2}(2n+1)\int_{-1}^{1}{\hat{\bar{\;p}}}_{m}(\zeta ,\omega )P_{n}(\zeta )\;d\zeta ,\;b_{v}^{\left(n\right)}(\omega )=\frac{1}{2}(2n+1)\int_{-1}^{1}{\hat{\bar{\;p}}}_{v}(\zeta ,\omega )P_{n}(\zeta )\;d\zeta .$$

Hence, we have, applying the inverse Fourier transform $F^{-1}$ to (5.4) and (5.5)

5.11 $$p_{m}^{\left(0\right)}(r,\zeta ,t)=\sum_{n=0}^{\infty }F^{-1}\left[c_{1}^{\left(n\right)}(\omega )r^{n}+\frac{M_{m}(\omega ){\tilde{A}}_{n}(\omega )}{M(\omega )}\frac{1}{\sqrt{r}}I_{n+(1/2)}\left(\sqrt{M(\omega )}r\right)\right]P_{n}(\zeta )$$

and

5.12 $$p_{v}^{\left(0\right)}(r,\zeta ,t)=\sum_{n=0}^{\infty }F^{-1}\left[d_{1}^{\left(n\right)}(\omega )r^{n}-\frac{M_{v}{\tilde{A}}_{n}(\omega )}{M(\omega )}\frac{1}{\sqrt{r}}I_{n+(1/2)}\left(\sqrt{M(\omega )}r\right)\right]P_{n}(\zeta ).$$

## 6. Cell problem numerical simulations

In this section, we discuss the numerical simulations used to find the solutions of the cell problems (3.29), (3.30), (3.41) and (3.42). We can see the geometry of the cell problems using the lymph node physiological data found in [1, appendix B] and summarized in table 1 in figure 1.

As we mentioned in §5, we suppose ***u***_*m*,0_ = **0**, ***f***_*m*_ = **0**, ***f***_*v*_ = **0**, which means that the solutions of the problems (3.30) and (3.42) are zeros. Moreover, we suppose that both porous media are isotropic, which means

6.1 $${\bar{K}}_{v}={\bar{K}}_{v}I=\frac{d^{2}}{\mu }{\left\langle K_{v}+K_{v}{\left(\nabla_{y}h_{v}\right)}^{T}\right\rangle }_{\Omega_{v}}I\;\mathrm{and}\;{\hat{\bar{K}}}_{m}(\omega )={\hat{\bar{K}}}_{m}(\omega )I=\frac{d^{2}}{\mu }{\left\langle {\hat{Q}}_{m}(y,\omega )\right\rangle }_{\Omega_{m}}I.$$

For the solution of the cell problem in the phase *Ω*_*v*_, we refer to [1, appendix C]. For the phase *Ω*_*m*_, we solve the problem (3.41) with *α* = 1 using the steady Brinkman equation module in COMSOL Multiphysics. We use the PARDISO solver and the $P_{2}^{3}-P_{1}$ finite-element discretization for the velocity and the pressure, respectively. Moreover, we perform a parametric sweep analysis varying *ω* to obtain the solution for different frequencies. After that, we apply the average operator to obtain ${\left\langle {\hat{Q}}_{m}(y,\omega )\right\rangle }_{\Omega_{m}}$. We obtain the dimensional hydraulic conductivity ${\hat{\bar{K}}}_{m}(\omega )$ shown in figure 2.

**Figure 3.**
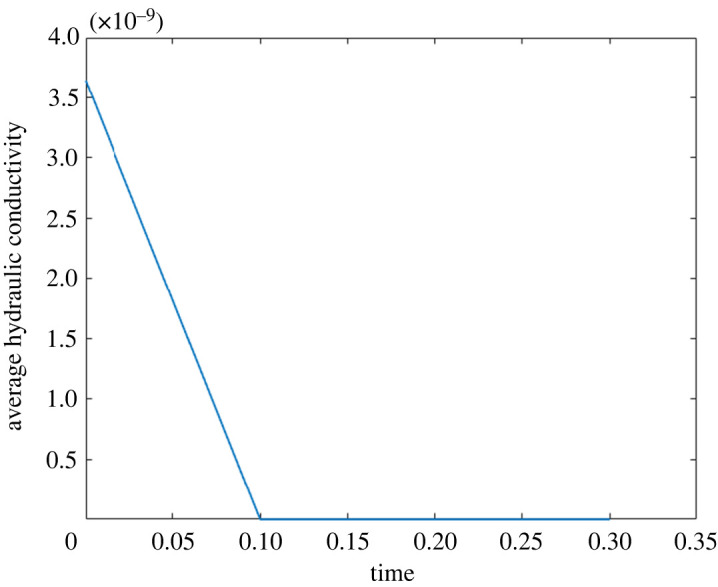
The inverse Fourier transform of the dimensional average hydraulic conductivity in ${\text{mm}}^{3}\;\text{s}\;{\text{mg}}^{-1}$ calculated numerically using the command ifft to the solution plot in figure 2 with physiological values of the lymph node (table 1).

If we take the values of the permeability ${\left\langle {\hat{Q}}_{m}(y,\omega )\right\rangle }_{\Omega_{m}}$ and we perform an inverse Fourier transform (with the Matlab built-in command *ifft*), we find the dimensional average hydraulic conductivity shown in figure 3. As expected, we have a permeability that decreases in time [8]. The decreasing imaginary part of ${\left\langle {\hat{Q}}_{m}(y,\omega )\right\rangle }_{\Omega_{m}}$ in figure 2 is connected to the decrease in time of the permeability (due to the memory effect of the macroscopic Darcy’s Law). If we compare the ifft result with the solution of the cell problem (3.45), we have a maximum relative error of 0.188%. Moreover, we have that the real part of the permeability is similar to the one found in the steady case in [1]. We believe these values to be realistic because, at time zero, we have the same permeability values we found in the steady setting [1]. Additionally, as time increases, the permeability tends to zero very fast, reflecting its diminishing influence on the flow dynamics at the time we are studying our flow, a characteristic commonly observed in memory-type equations [8].

To study in more detail the mesh of the previous solution, we perform an adaptive mesh refinement study. After this process, we compare the two ${\hat{\bar{K}}}_{m}(\omega )$ that we found, and we obtain, for all the values of *ω*, a maximum relative error of 0.06%.

To test the Brinkman equation module in Comsol with complex numbers, we run some simulations in a cylindrical geometry and we compare the solution to the following explicit solution (found in [1]):

$$W_{33,v}^{DB}(r)=K^{*}\left[1-\frac{J_{0}\left(i\sqrt{\left(1/\mu^{*}K^{*}\right)}r\right)}{J_{0}\left(i\sqrt{\left(1/\mu^{*}K^{*}\right)}{\hat{r}}_{c}\right)}\right],\;W_{31,v}^{DB}=W_{32,v}^{DB}=0.$$

Taking the average, we obtain

6.2 $${\left\langle W_{33,v}^{DB}\right\rangle }_{\Omega_{v}}=\frac{2\pi l_{c}}{\left|\Omega_{\text{cyl}}\right|}K^{*}\left[\frac{{\hat{r}}_{c}^{2}}{2}+i\sqrt{\mu^{*}K^{*}}{\hat{r}}_{c}\frac{J_{1}\left(i\sqrt{\left(1/\mu^{*}K^{*}\right)}{\hat{r}}_{c}\right)}{J_{0}\left(i\sqrt{\left(1/\mu^{*}K^{*}\right)}{\hat{r}}_{c}\right)}\right],$$

where *l*_*c*_ is the cylinder length, *Ω*_cyl_ is the cylinder volume, K* is the permeability, ${\hat{r}}_{c}$ is the cylinder radius, μ* is the viscosity, *J*_0_ and *J*_1_ are the Bessel functions of the first and second kind, respectively. We run the numerical simulations and compare the solution to the explicit one (6.2) with the following (non-dimensional) data: *l*_*c*_ = 1, ${\hat{r}}_{c}$ = 7.7 × 10^−3^, K* = 6.66 × 10^−6^ + *i*, 6.66 × 10^−6^ + 10*i*, 6.66 × 10^−6^ + 100*i*, μ* = 1. We found that the maximum relative error between the numerical and the explicit solution is 0.67% for the real part and 1.9% for the imaginary part.

## 7. Study of the fluid flow in a lymph node

In this section, we study the fluid flow inside the lymph node using the model results obtained in the previous sections of this work. In particular, we use the explicit solution of §5 applied to the porous region of the node (the LC) because all the lymph node vascularization is situated in this region [47–49]. Moreover, we assume that the lymph node has a spherical shape, and we use physiological lymph node data inspired by a mouse popliteal lymph node [1,47,59]. The explicit solution of §5 is implemented in Matlab. To compute the inverse Fourier transform, we use the Matlab built-in command *ifft*, that is, the *inverse fast Fourier transform*.

We have that *Ω*_*v*_ and *Ω*_*m*_ are the portions of the domain that represent the blood vessels and the FRC network, respectively. Choosing $\bar{\;p}(t)=\sigma (\pi_{m}(t)-\pi_{v}(t))$, we describe the fluid exchange between the phases mentioned above using the well-known Starling equation [62,63]. Moreover, all the physiological data used are summarized in table 1, and the meaning of these data is explained in [1, appendix B].

As we can see from the previous sections, here the hydraulic conductivity of the phase *Ω*_*m*_ depends on time due to the memory term in the Darcy equation (4.1). This is very different from the previous works on the lymph node [1,47,53,54,57–59], as it allows for a comprehensive analysis of the lymph’s time behaviour through a rigorous homogenization method.

Using physiological data obtained from the literature on the lymph node, we find that the parameter that governs the time dependency of our problem is *η* ≈ 0.1, in line with the Womersley number found in the lymphatic system [36].

At the boundary, we have the following boundary conditions:

$$p_{m}(R,\zeta ,t)={\bar{\;p}}_{m}(\zeta ,t)\;\mathrm{and}\;p_{v}(R,\zeta ,t)={\bar{\;p}}_{v},$$

where ${\bar{\;p}}_{m}(\zeta ,t)$ is a general function in *ζ* and *t*, and ${\bar{\;p}}_{v}$ is the mean of the blood vessel pressure data (in general constant).

As we mentioned in [1], the variation with respect to ζ=cos⁡θ of the boundary condition ${\bar{\;p}}_{m}$ is essential to mimic the pressure distribution in the SCS. The precise pressure distribution of the SCS is not well known so, to circumvent this lack of data in the literature and inspired by [45], we take a linear relation between the pressure and ζ=cos⁡θ. This linear relation connects the maximum value of the pressure ${\bar{\;p}}_{m,\max}=3.9\;\mathrm{mmHg}\approx 5.2\times 10^{5}\;\mathrm{mPa}$ with the minimum value of ${\bar{\;p}}_{m,\min}=3\;\mathrm{mmHg}\approx 4\times 10^{5}\;\mathrm{mPa}$, taken from the numerical results of [47]. Moreover, to describe the pulsatile inflow, we take a pressure distribution that varies over time in the following way:

7.1 $${\bar{\;p}}_{m}(\zeta ,t)={\bar{\;p}}_{m,\min}+\frac{1}{2}(1-\cos(\pi t))\frac{\zeta +1}{2}({\bar{\;p}}_{m,\max}-{\bar{\;p}}_{m,\min}).$$

**Figure 4.**
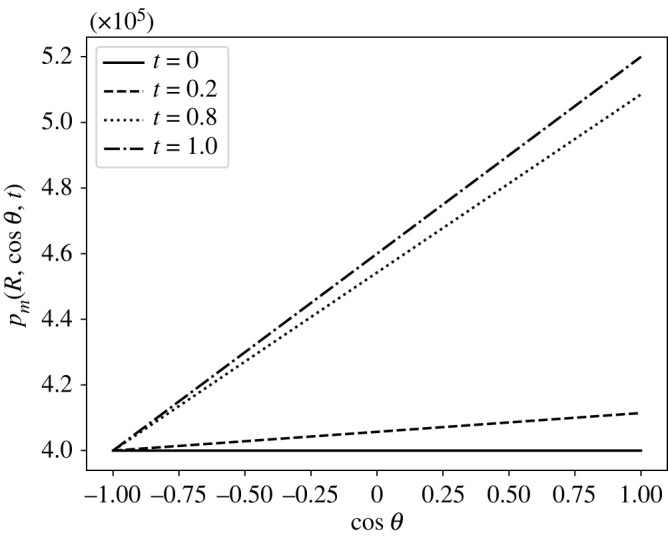
The pressure distribution in mPa of equation (7.1) calculated at different times (in seconds).

We can see the behaviour of the boundary condition (7.1) with respect to *ζ* at different times in figure 4.

**Figure 5.**
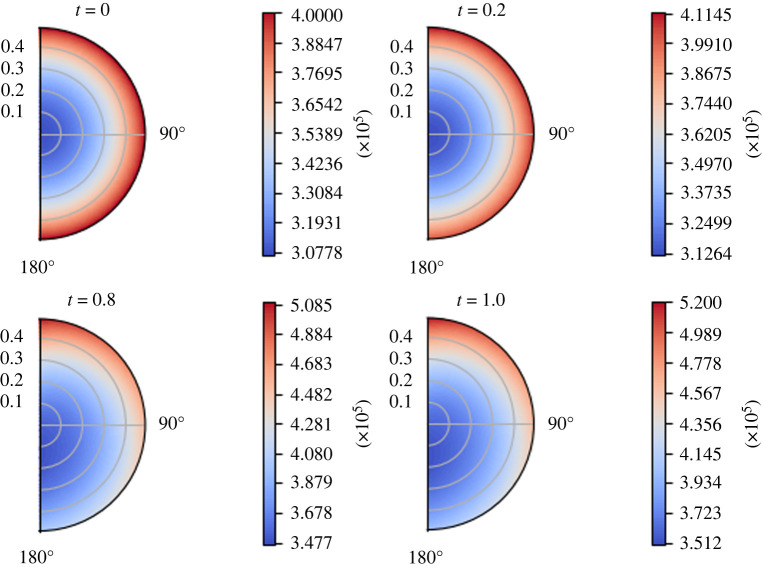
The variation of the interstitial pressure *p*_*m*_ (in mPa) with respect to time (in seconds), with the parameters in table 1 and *π*_*v*_ − *π*_*m*_ = 1.02 × 10^6^ mPa, *L*_*p*_ = 5.475 × 10^−10^ mm s^−1^ mPa^−1^, ${\bar{\;p}}_{v}=6.66\times 10^{5}\;\mathrm{mPa}$ and the boundary conditions (7.1).

We use the following physiological parameters: *σ* = 0.88, *π*_*v*_ − *π*_*m*_ = 1.02 × 10^6^ mPa, $L_{p}=5.475\times 10^{-10}\;\text{mm}\;s^{-1}{\text{ mPa}}^{-1}$ and ${\bar{\;p}}_{v}=6.66\times 10^{5}$. We can see the behaviour of the interstitial pressure *p*_*m*_ over time in figure 5. As we can see, the minimum of the interstitial pressure *p*_*m*_ increases and moves from the centre of the node to the lower part of the node. This is due to the fact that, as time passes, the boundary pressure distribution (7.1) remains linear but the maximum of the pressure increases, and this effect combines with the effect of the fluid exchange between phases. This phenomenon highlights the importance of the time dependency of the flow inside the node. We note that the Darcy equation linearly relates the fluid discharge to the pressure gradient, so the lymph moves accordingly to the pressure, which means that a sink term is represented by a lower pressure region in the node.

**Figure 6.**
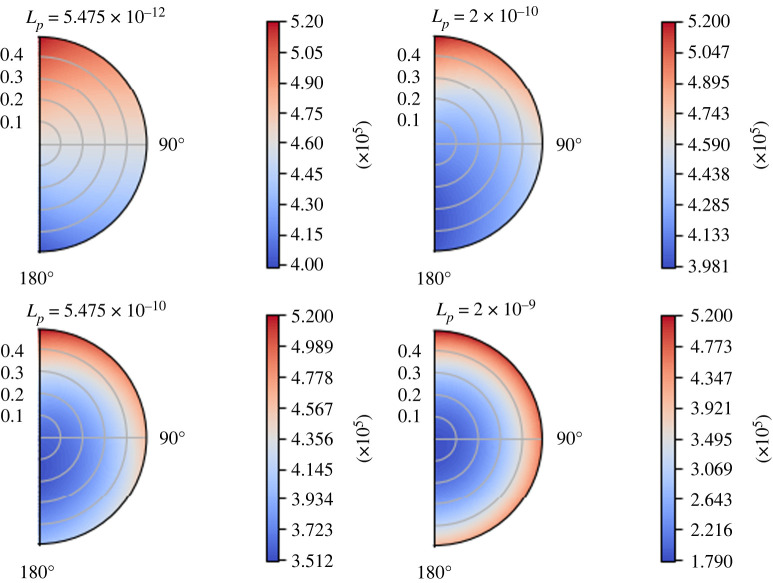
The variation of the interstitial pressure *p*_*m*_ (in mPa) varying *L*_*p*_ (in mm s^−1^ mPa^−1^) at time *t* = 1 s, with the parameters in table 1, *π*_*v*_ − *π*_*m*_ = 1.02 × 10^6^ mPa, ${\bar{\;p}}_{v}=6.66\times 10^{5}\;\mathrm{mPa}$ and the boundary conditions (7.1).

As expected and in accordance with [1], increasing *L*_*p*_ results in a decrease of *p*_*m*_ and an increase of *p*_*v*_ at the centre of the node. We can see this behaviour of the interstitial pressure *p*_*m*_ in the contour plot of figure 6 at time *t* = 1 s. As we can see, the results are in line with the steady one [1], which means that the increase of *L*_*p*_ decreases and moves the minimum of *p*_*m*_ towards the centre of the node.

**Figure 7.**
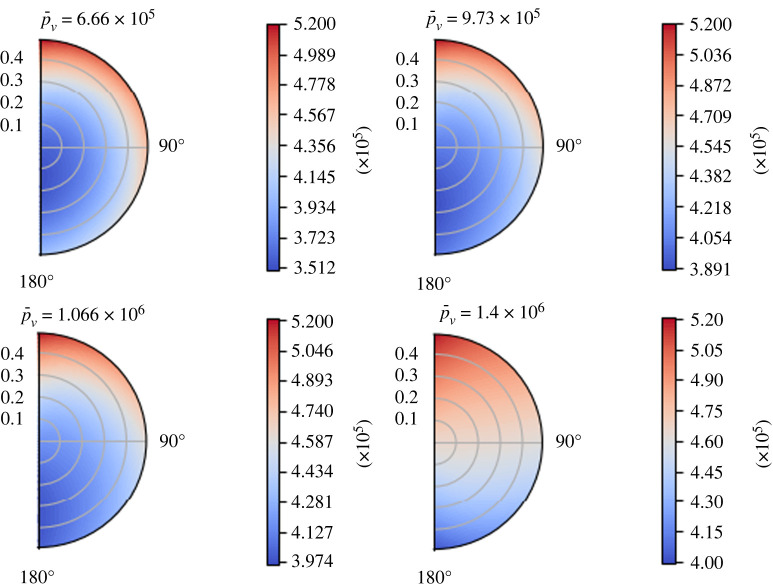
The variation of the interstitial pressure *p*_*m*_ (in mPa) varying the mean blood vessel pressure ${\bar{\;p}}_{v}$ (in mPa) at time *t* = 1 s, with the parameters in table 1, *π*_*v*_ − *π*_*m*_ = 1.02 × 10^6^ mPa, *L*_*p*_ = 5.475 × 10^−10^ mm s^−1^ mPa^−1^ and the boundary conditions (7.1).

**Figure 8.**
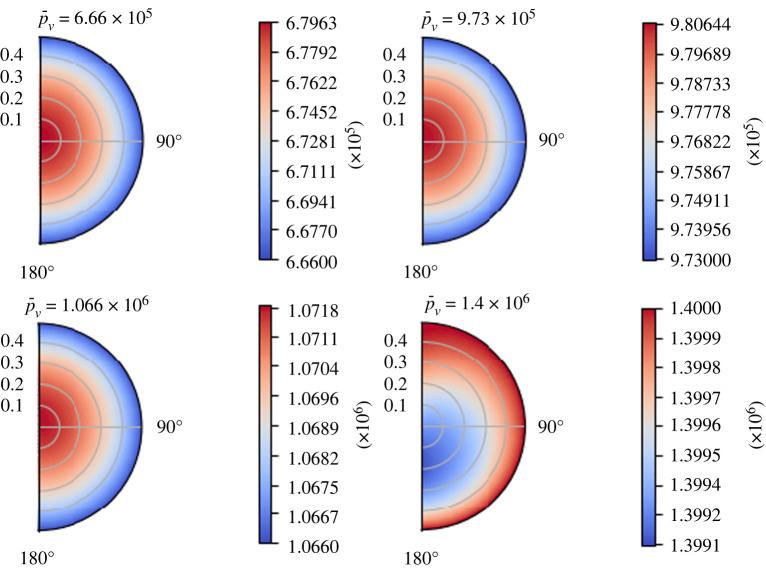
The variation of the blood vessel pressure *p*_*v*_ (in mPa) for some values of the mean blood vessel pressure ${\bar{\;p}}_{v}$ (in mPa) at time *t* = 1 s, with the parameters in table 1, *π*_*v*_ − *π*_*m*_ = 1.02 × 10^6^ mPa, *L*_*p*_ = 5.475 × 10^−10^ mm s^−1^ mPa^−1^ and the boundary conditions (7.1).

**Figure 9.**
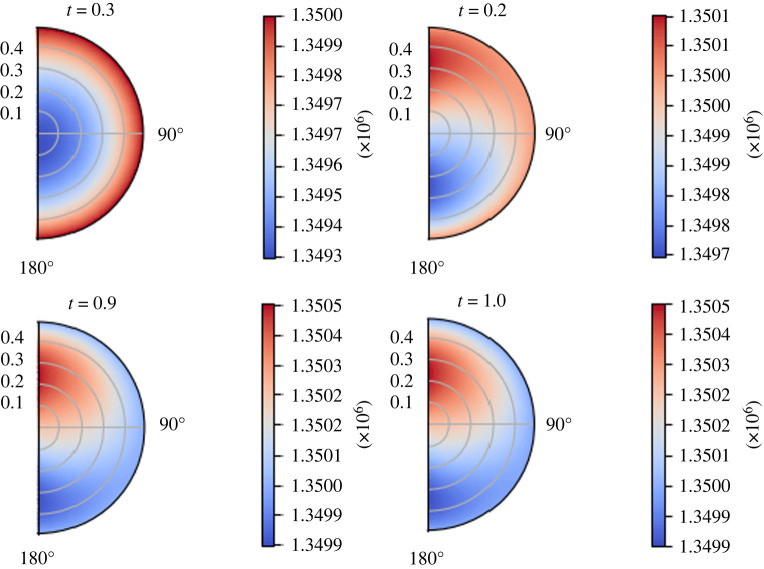
The variation of the blood vessel pressure distribution *p*_*v*_ (in mPa) with respect to time (in seconds), with the parameters in table 1, *π*_*v*_ − *π*_*m*_ = 1.02 × 10^6^ mPa, ${\bar{\;p}}_{v}=1.35\times 10^{6}\;\mathrm{mPa}$, *L*_*p*_ = 5.475 × 10^−10^ mm s^−1^ mPa^−1^ and the boundary conditions (7.1).

In figures 7 and 8, one can see the behaviour of the interstitial pressure *p*_*m*_ and the blood vessel pressure *p*_*v*_ for some values of ${\bar{\;p}}_{v}$ at time *t* = 1 s. When ${\bar{\;p}}_{v}$ increases, we have the opposite behaviour of the one found for *L*_*p*_: the minimum value of the interstitial pressure *p*_*m*_ moves towards the lower part of the node and the fluid exchange between the two phases decreases (because the minimum of *p*_*m*_ increases); on the contrary, the maximum of the blood vessel pressure *p*_*v*_ increases. We have an inversion of the flow direction for every *t* at ${\bar{\;p}}_{v}\approx 1.4\;\text{mPa}\approx 10.5\;\text{mmHg}$, which is the same value found in [47] and in [1]; from this value to higher values of ${\bar{\;p}}_{v}$, the maximum of *p*_*m*_ starts to move towards the centre of the node. This behaviour is in accordance with the findings in [1]. Moreover, we can have flow inversion for only certain values of the time; for instance, we can see figure 9, where we plot the blood vessel pressure *p*_*v*_ at different times with ${\bar{\;p}}_{v}=1.35\times 10^{6}\;\mathrm{mPa}$. For the initial times, we have a flow inversion, which means that the fluid moves from phase *Ω*_*v*_ to phase *Ω*_*m*_, resulting in a region of minimum pressure at the centre of the node for *p*_*v*_. As time passes, the boundary condition (7.1) increases the pressure in the upper region of the lymph node (near *θ* = 0), and this results in a region of higher pressure zone near *θ* = 0 and the region of lower pressure zone moves near *θ* = *π*.

**Figure 10.**
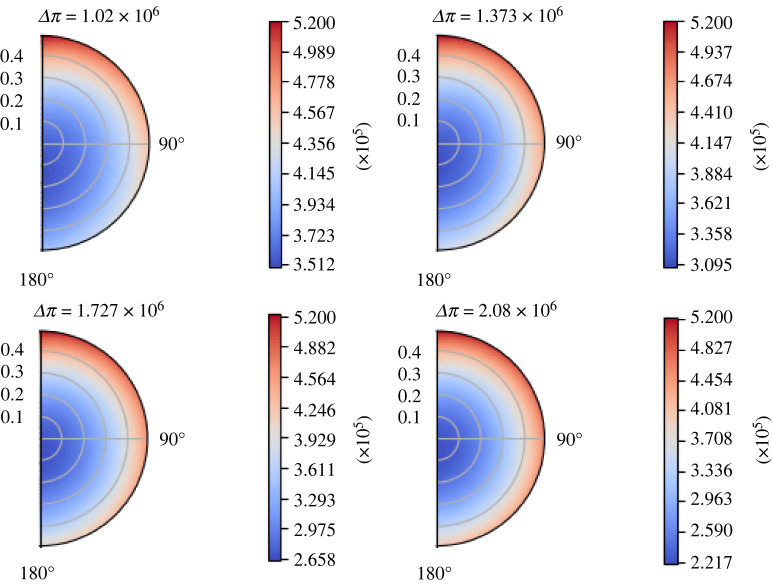
The variation of the interstitial pressure *p*_*m*_ (in mPa) varying Δ*π* (in mPa), with the parameters in table 1, ${\bar{\;p}}_{v}=6.66\times 10^{5}\;\mathrm{mPa}$, *L*_*p*_ = 5.475 × 10^−10^ mm s^−1^ mPa^−1^, and the boundary conditions (7.1).

Increasing Δ*π* means an increase in the concentration difference between the blood vessels and the FRC phase, and this results in a decrease of the minimum of *p*_*m*_ and moves it to the centre of the node, and an increase of the maximum of *p*_*v*_. We can see a contour plot of *p*_*m*_ at *t* = 1 s varying Δ*π* in figure 10.

**Figure 11.**
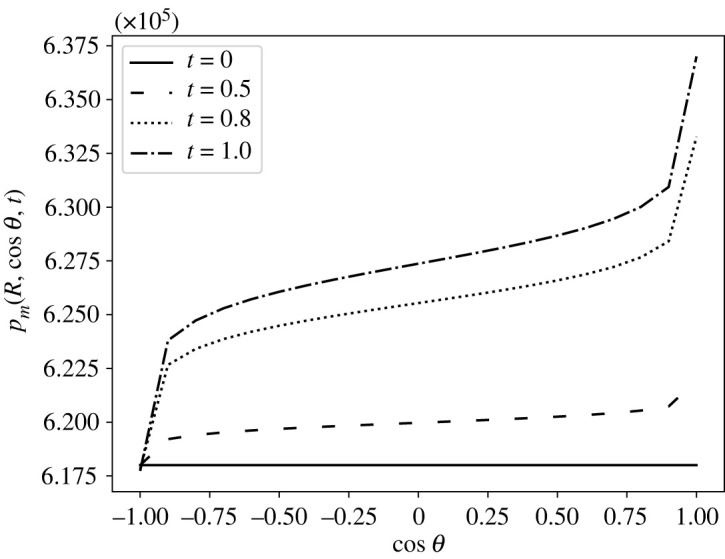
The variation of the interstitial pressure *p*_*m*_ (in mPa) calculated at the boundary (using the computations of [59]) with respect to ζ=cos⁡θ at different times (in seconds), with the parameters in table 1, Δ*π* = 1.02 × 10^6^ mPa, $L_{p}=5.475\times 10^{-11}\;\text{mm}\;s^{-1}\;{\mathrm{mPa}}^{-1}$ and ${\bar{\;p}}_{v}=1.06\times 10^{6}\;\mathrm{mPa}$.

**Figure 12.**
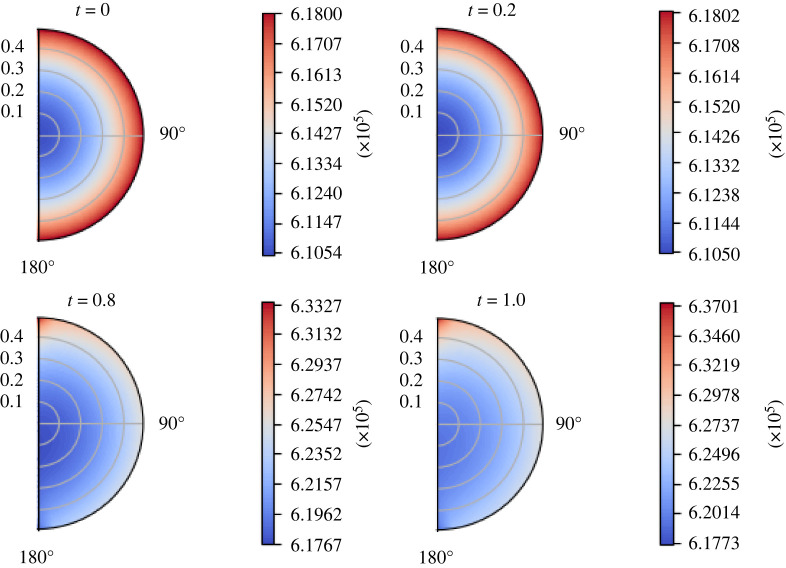
The variation of *p*_*m*_ (in mPa) with respect to time (in seconds) with the boundary pressure shown in figure 11, Δ*π* = 1.02 × 10^6^ mPa, *L*_*p*_ = 5.475 × 10^−11^ mm s^−1^ mPa^−1^, ${\bar{\;p}}_{v}=1.06\times 10^{6}\;\mathrm{mPa}$ and the parameters in table 1.

**Figure 13.**
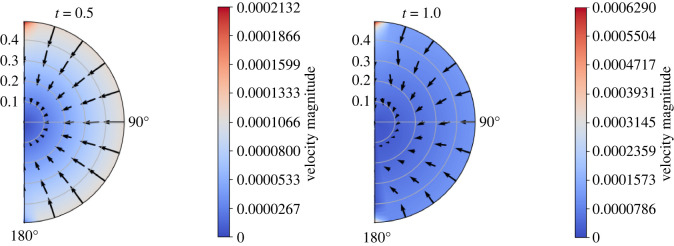
The contour plot of the velocity of the phase *Ω*_*m*_ (in mm s^−1^) with respect to time (in seconds) with the boundary pressure shown in figure 11, Δ*π* = 1.02 × 10^6^ mPa, *L*_*p*_ = 5.475 × 10^−11^ mm s^−1^ mPa^−1^, ${\bar{\;p}}_{v}=1.06\times 10^{6}\;\mathrm{mPa}$ and the parameters in table 1.

From the fact that the boundary condition ${\bar{\;p}}_{m}(\zeta ,t)$ can be chosen as we want, we solve our explicit solution using the solution we found in our previous paper [59] using the stream function approach. We refer to [59] for more details about the computations and the data of this pressure distribution; this solution is calculated in a div-free setting, where we fix ${\bar{\;p}}_{m}(R_{2},-1)=6.18\times 10^{5}\;\mathrm{mPa}$ and with a time pulsation of the form (1−cos⁡(πt))/2. In figure 11, we can see the boundary pressure distribution. The fast increment near *ζ* = 1 represents the inlet condition, and the fast decrement near *ζ* = −1 represents the outlet condition. In figures 12 and 13, we can see the pressure and the velocity distribution over time using the parameters ${\bar{\;p}}_{v}=1.06\times 10^{6}\;\mathrm{mPa}$, *π*_*v*_ − *π*_*m*_ = 1.02 × 10^6^ mPa, *L*_*p*_ = 5.475 × 10^−11^ mm s^−1^ mPa^−1^, and the above-mentioned boundary pressure. As we can see, for initial times, we have a similar pressure distribution to that we found in the previous case but, as time passes, it becomes more evident the higher pressure near the inlet and the lower pressure near the outlet. We can see this behaviour for the velocity magnitude too, where at *t* = 1 s, we can see a higher difference between the inlet–outlet velocity and the velocity at the centre of the node.

Moreover, we have that the velocities that we found inside the LC are in agreement with the literature [47,55,79–81]. We found a higher velocity (at time *t* = 1 s) with respect to the steady case [1]; moreover, we have that the pressure change (at time *t* = 1 s) between the upper region (near the inlet condition) and the lower region (near the outlet condition) is higher here too.

Finally, we have performed a parameter sensitivity analysis and found that on varying the average hydraulic conductivity, the resulting pressure (interstitial and blood pressure) exhibits only a relatively small variation. In particular, a variation of 1, 2, and 5% of the average hydraulic conductivity corresponds to the relative variations of the interstitial pressure of 0.012, 0.0238 and 0.0578%.

## 8. Conclusion

In this paper, we use the asymptotic homogenization technique in a time-dependent setting, starting from the equations and interface conditions (2.1), (2.2), (2.4), assuming both local periodicity and macroscopic uniformity.

This model is a non-trivial extension of our previous model [1]; here, we have considered a time-dependent Darcy–Brinkman equation, which results in a Darcy equation with memory at the macroscale for the phase *Ω*_*m*_. The time dependency is considered for the pulsation behaviour of the lymph [36,37,61,82] and allows us to study the fluid behaviour inside the lymph node in more detail [39,40,59]. With lymph node physiological data, the characteristic time is *η* ≈ 0.1: this is in agreement with the Womersley number found for the lymphatic system [36].

This model has been designed to be applied to the flow of lymph within the lymph node, but the derivation of the model has been intentionally kept as general as possible to be applicable to other problems as well.

After the derivation of the macroscopic equations that described the time-dependent fluid flow (§4), in §5, we have found the explicit solution of the proposed model in a spherical domain, using the computations of the solution that we found in [1] and the properties of the Fourier transform. Subsequently, we have studied the fluid and pressure distribution within the lymph node using the above-mentioned explicit solution and physiological data inspired by an idealized spherical mouse popliteal lymph node. Incorporating the temporal component into the proposed model has allowed us to study how lymph pulsations influence these quantities within a lymph node. Regarding this multiscale formulation, our primary focus has been directed towards the porous region of the lymph node (the LC). Moreover, we have placed particular emphasis on studying the fluid exchange that occurs between the interstitial space of the lymph node and the blood vessels, which are exclusively present in this specific part of the node [47–49]. We have analysed how various parameters influence fluid absorption and pressure (i.e. velocity) over time, and the obtained results align with findings documented in the literature [1,47,73–75,83].

Let us now explore considerations that could enhance the model in the future. In this work, we solely focused on the LC, but it would be interesting to couple this model with the fluid flow in the SCS to describe the fluid flow in the entire lymph node.

The Beavers–Joseph–Saffman interface condition (2.4) used in this work is initially established in a two-dimensional setting, and expanding it to three dimensions presents a significant and complex challenge [2,84–88]. Moreover, it would be interesting to study how the physico-chemical properties of the interface affect the solution [67].

When we applied our model to the lymph node, we assumed negligible forces to simplify our model and due to a lack of data regarding them in the literature. However, it is important to note that in practical situations, such forces can play a significant role, especially when using electromagnetic fields, as seen in [89,90] within the context of cancer hyperthermia. Hence, when we have access to physiological data, it becomes crucial to account for the impact of inhomogeneous volume loads, as discussed in [24]. We can say the same for the velocity initial condition, which we have assumed to be zero. In general, we can have more general fluid behaviour as an initial condition, in particular in drug delivery applications [25].

In our model, we have taken into account the time variation of the concentration of protein inside the node, but we have supposed it to be constant due to a lack of precise data. Incorporating the temporal and spatial behaviour of protein concentration, as discussed in [3], and together with physiological data, would be a compelling and valuable direction to explore.

In this work, we have assumed a rigid porous matrix to keep the model as simple as possible and due to the lack of biological data regarding this problem; a possible extension of this model is to take into account a deformable matrix that interacts with the lymph flow inside the node, for example by considering the modelling framework developed in [91,92].

Finally, due to our explicit analysis, we assumed a spherical lymph node. In general, lymph nodes have a more complex geometry, typically an ellipsoid shape [47,53,54]. If we can access more realistic data regarding their shape through medical imaging [53,54], our model could be used for more realistic numerical simulations to make physiologically meaningful predictions in the future.

## Data Availability

This article has no additional data.
